# Gas and seismicity within the Istanbul seismic gap

**DOI:** 10.1038/s41598-018-23536-7

**Published:** 2018-05-01

**Authors:** L. Géli, P. Henry, C. Grall, J.-B. Tary, A. Lomax, E. Batsi, V. Riboulot, E. Cros, C. Gürbüz, S. E. Işık, A. M. C. Sengör, X. Le Pichon, L. Ruffine, S. Dupré, Y. Thomas, D. Kalafat, G. Bayrakci, Q. Coutellier, T. Regnier, G. Westbrook, H. Saritas, G. Çifçi, M. N. Çağatay, M. S. Özeren, N. Görür, M. Tryon, M. Bohnhoff, L. Gasperini, F. Klingelhoefer, C. Scalabrin, J.-M. Augustin, D. Embriaco, G. Marinaro, F. Frugoni, S. Monna, G. Etiope, P. Favali, A. Bécel

**Affiliations:** 1 Ifremer, Département Ressources Physiques et Ecosystèmes de fond de Mer (REM), Plouzané, F-29280 France; 2 CEREGE, Aix Marseille Univ., CNRS, IRD, INRA, Coll. France, Aix-Marseille, France; 3 Lamont-Doherty Earth Observatory, Palisades, NY USA; 4 Universidad de los Andes, Bogotà, Colombia; 5 ALomax Scientific, 06370 Mouans-Sartoux, France; 6 Kandilli Observatory and Earthquake Research Institute, Boğaziçi University, Istanbul, Turkey; 7 Istanbul Technical University, Istanbul, Turkey; 8 Ocean and Earth Science, National Oceanography Centre, Southampton, UK; 9 School of Geography, Earth and Environmental Sciences, University of Birmingham, Birmingham, UK; 10 Mineral Research & Exploration General Directorate, MTA, Ankara, Turkey; 11 Institute for Marine Science and Technology, Dokuz Eyiul Universitesi, Izmir, Turkey; 12 Helmholtz-Centre Potsdam German Centre for Geosciences GFZ, Section 4.2 Geomechanics and Rheology, Telegrafenberg, 14473 Potsdam, Germany; 13 Freie Universität Berlin, Department of Earth Sciences, Malteser Strasse 74-100, 12249 Berlin, Germany; 14 Institute of Marine Science, ISMAR-CNR, Bologna, Italy; 15 Istituto Nazionale di Geofisica e Vulcanologia, INGV, Roma, Italy; 16 Faculty of Environmental Science and Engineering, Babes-Bolyai University, Cluj-Napoca, Romania

## Abstract

Understanding micro-seismicity is a critical question for earthquake hazard assessment. Since the devastating earthquakes of Izmit and Duzce in 1999, the seismicity along the submerged section of North Anatolian Fault within the Sea of Marmara (comprising the “Istanbul seismic gap”) has been extensively studied in order to infer its mechanical behaviour (creeping vs locked). So far, the seismicity has been interpreted only in terms of being tectonic-driven, although the Main Marmara Fault (MMF) is known to strike across multiple hydrocarbon gas sources. Here, we show that a large number of the aftershocks that followed the M 5.1 earthquake of July, 25^th^ 2011 in the western Sea of Marmara, occurred within a zone of gas overpressuring in the 1.5–5 km depth range, from where pressurized gas is expected to migrate along the MMF, up to the surface sediment layers. Hence, gas-related processes should also be considered for a complete interpretation of the micro-seismicity (~M < 3) within the Istanbul offshore domain.

## Introduction

Since 1939, the North Anatolian Fault (NAF) -one of the most active strike-slip faults on Earth, like for instance the San Andreas Fault or the Altyn Tagh Fault- has produced an unique sequence of M > 7 earthquakes, starting from eastern Anatolia and propagating to the west towards Istanbul^1–3^. Prior to this sequence, which ended in 1999 with the devastating earthquakes of Izmit and Duzce, causing more than 20000 casualties at the eastern end of the Sea of Marmara, the fault ruptured in 1912. This earlier rupture developed in the west at the transition into the North Aegean, leaving the Marmara section of the NAF as the only part of the fault not being activated since 1766. The Istanbul-Marmara region between the 1912 and 1999 ruptures is thus generally considered to represent a seismic gap with an earthquake potential^4,5^ of M up to 7.4. Intensive surveys have been conducted since 1999 to investigate the main branch of the NAF below the Sea of Marmara to better understand its seismotectonic setting and the resulting seismic hazard for the densely populated (>15 million) greater Istanbul region.

**Figure 1:**
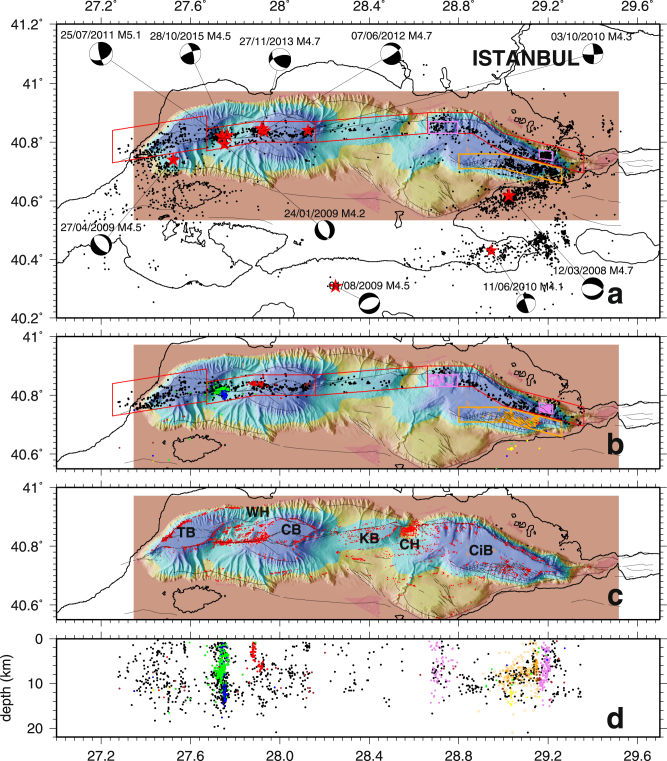
Seismicity (after ref.^8^) and gas emissions within the Sea of Marmara. (**a**) Full seismicity map from 2007 to 2012 based on the recordings from 132 land stations, as well as from temporary seabottom networks, including 5 cabled observatories from 2009 to 2011 and 10 autonomous stations deployed by Ifremer from april 2011 to july 2011 (see location of stations in ref.^8^). The Western Sea of Marmara is regularly affected by earthquakes of magnitude >4.5. The two earthquakes of magnitude >4.0 which occurred in 2013 and 2015 were therefore added to complete the general pattern of seismicity in this area (locations from KOERI catalog). For all maps, thin black lines indicate major structural features, from ref.^3^. (**b**) Selected micro-earthquakes with epicentres located within the boxes shown in panel A, respectively centered along the MMF and within the southern part of the Cinarçik Basin. Coloured dots indicate those aftershocks that occurred within the Western Sea of Marmara, 7 days after the earthquakes of magnitude >4 (years 2007 to 2012 only). Blue: 27/04/2009; pink: 03/10/2010; red: 25/07/2011; green: 07/06/2012. (**c**) Sites of acoustically detected gas emissions (red dots), from ref.^22^. TB: Tekirdag Basin. WH: Western High; KB: Kumburgas Basin; CH: Central High; CiB: Cinarçik Basin. Note that in the Central High and the in the Kumburgas basins, gas is not found within the Main Marmara Fault Valley, nor along the Fault trace, but on top of adjacent structures or at the edge of basins (see refs^21,22^). (**d**) Depth of selected earthquakes plotted versus longitude, with events from aftershock sequences represented using the same colour code as in panel B. Orange dots indicate events from the area delineated by the orange box (Panel B) in the South Cinarçik Basin; Violet dots are for events from the area delineated by the 2 violet boxes shown in Panel B. Color bar for bathymetry is displayed in Fig. 2. All panels were created with GMT software, Version 4.5.11.

Geological and geophysical, marine surveys since 2000 have revealed the geometry of the submarine Main Marmara Fault (MMF) system^6,7^. Seismological studies have shown that the seismicity along the MMF exhibits a strong lateral variability^8–14^. Seismicity in the Sea of Marmara is unevenly distributed and concentrates in spatial and temporal clusters^8,11^. In the western part of the MMF (e.g. the Tekirdag Basin, the Western High and the Central Basin) (Fig. 1), seismicity appears to be localized in several restricted active zones, while the central part, which encompasses the Kumburgaz Basin and the Central High, is comparatively seismically silent. In the Cinarcik Basin, offshore Istanbul, at the western termination of the 1999 Izmit rupture, the installation of seismometer arrays on the Prince Islands at 2–3 km from the main fault led to the identification of a 30 by 8 km aseismic patch that was interpreted as a locked zone on the MMF^11^. A major aspect of seismic hazard assessment is to determine the mechanical behaviour of these potentially dangerous locked segments, which may rupture during the next expected Marmara earthquake. The presence of the water cover above the fault trace and the absence of islands to the south of the fault limits the use of GPS data in estimating the strain accumulation and slip deficit along each of the segments^15^. Efforts are presently on the way to collect acoustic-based geodetic data and encouraging results have recently been obtained^16^. Still, the detailed analysis of micro-seismicity maps remains critical.

To date, the micro-seismicity along the MMF has always been interpreted strictly for its tectonic origin (as a result of fault deformation mode and behaviour), while the potential role of gas-related processes to induce shallow micro-seismicity has been disregarded, although gas (generally methane) is known to be widespread on the Marmara seafloor^17–23^. In the Western Sea of Marmara, events of magnitude greater than ~ 4.2 regularly occur, generating large sequences of aftershocks, that appear to be vertically distributed below sites where a high density of gas seeps have been identified along the MMF^21,22^. “Vertical swarms dramatically appear in the recently published map of relocated earthquakes^8^ from 2007 to 2012, particularly for the aftershocks that followed the M_L_ 4.2, 4.7 and 5.1 events of 24/01/2009, 25/07/2011 and 07/06/2012, which all occurred where the density of gas emissions is maximum^16,17^ (in Fig. 1, note that along the Central High and Kumburgas fault segments, gas emissions are not found within the fault valley, but on adjacent structures, for instance on top of the Central High). However, the seismicity map shown in Fig. 1 is based on a 1D velocity model^10^, that fits to the velocity structure onshore but not to the one from the deep offshore. This probably has severe implication for the precise earthquake depth determination, particularly for shallow seismicity.

Hence, before any further analysis can be conducted, improved depth determinations of shallow earthquakes are needed. Here, we present new, high-resolution location results for the sequence of aftershocks that followed the M_L_ 5.1 earthquake that stroke on July 25^th^, 2011 at a depth of ~11–14 km^24^ below the “Western High”, a sedimentary anticline structure -up to ~7 to 8 km thick- where gas emissions, associated with traces of oil and gas hydrates, have been sampled^23^. The sequence of aftershocks (550 events detected in total) was monitored by a local network of two permanent cabled Ocean Bottom Seismometers (OBS) operated by KOERI and nine temporary autonomous stations deployed by IFREMER^25^. Unfortunately, the OBS located right above the hypocentral region failed a few days before the mainshock, limiting the depth-resolution. To improve the depth resolution, P- and S-wave arrivals were all manually checked to control the pick quality and to avoid misleading picks due to micro-events produced by gas expulsion at the seabed^26,27^. Then, special care was given to the velocity model. Since the basins of the Sea of Marmara are filled with more than 5 km of Plio-Quaternary soft (“slow”) sediments, the seismic velocity structure offshore is drastically different from the one onshore. For instance, based on deep-penetration, multi-channel seismic data^28^, the P-wave velocities were reported to be very low for the sea-bottom deposits, especially in the deep bathymetric trough (1.6 to 1.8 km/s) and gradually increasing from the sea-bottom to the pre-kinematic basement, where they reach values of 4 to 4.2 km/s. Additional data provided by OBS wide-angle reflection and refraction seismics^13^ indeed indicate that P-wave velocities in troughs do not exceed 2 km/s within the first kilometer below seafloor (bsf) nor 2.5 km/s between 2 and 3 km bsf. We thus tested a number of velocity models, 1D and 3D encompassing either the whole Marmara region^29,30^ or the deep, sub-marine domain *stricto-sensu*^25,31^.

**Figure 2:**
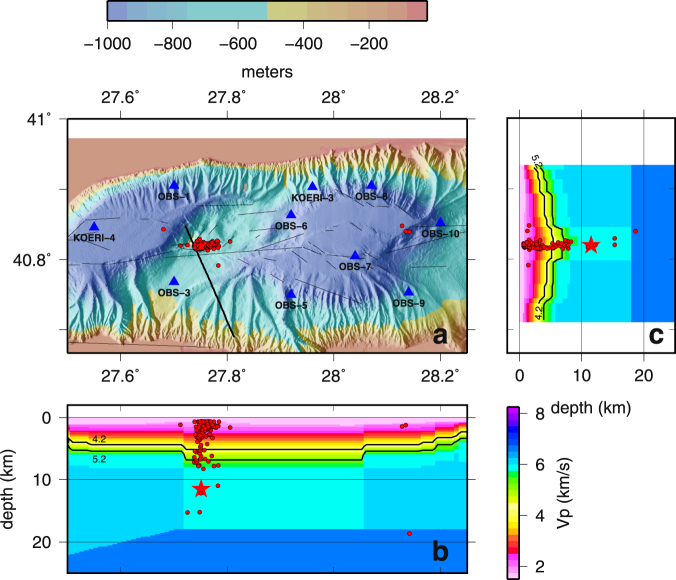
Panel a displays hypocenter relative locations (red dots) for the aftershocks that followed the M 5.1 earthquake of July 25, 2011. Locations were obtained using manual picks and the high-resolution P-wave velocity model described in Appendix 1. Triangles indicate OBS locations. Thick black line indicates the seismic profile collected in 2001 during the Seismarmara Cruise^28,36^ shown in Fig. 5. Thin black lines are for active faults^7^. Panels b anc c display the cross-sections of depth below sea-level along an East-West line crossing at 40.80°N and along a North-South line crossing at 27.78°E, respectively, with the aftershocks and the velocity structure extracted from the P-wave velocity grid (Appendix 1). The two iso-velocity lines at 4.2 and 5.2 km/s correspond to the syn-kinematic and post-kinematic basement, respectively^28,31^. Red star indicates the absolute location of the M_w_ 5.1 earthquake (the position obtained with land and sea-bottom stations is consistent within 0.5 km with the position obtained using OBSs only). First, absolute locations of all the events of the aftershock sequence were obtained using the NonLinLoc^33^ software package. Then relative locations were computed using NonLDiffLoc^33^ on a selection of “well-constrained” events, e.g.: number of stations ≥ 7; RMS < 250 msec; gap ≤150°; error in depth smaller than z. Image created with GMT software, Version 4.5.11.

**Figure 3:**
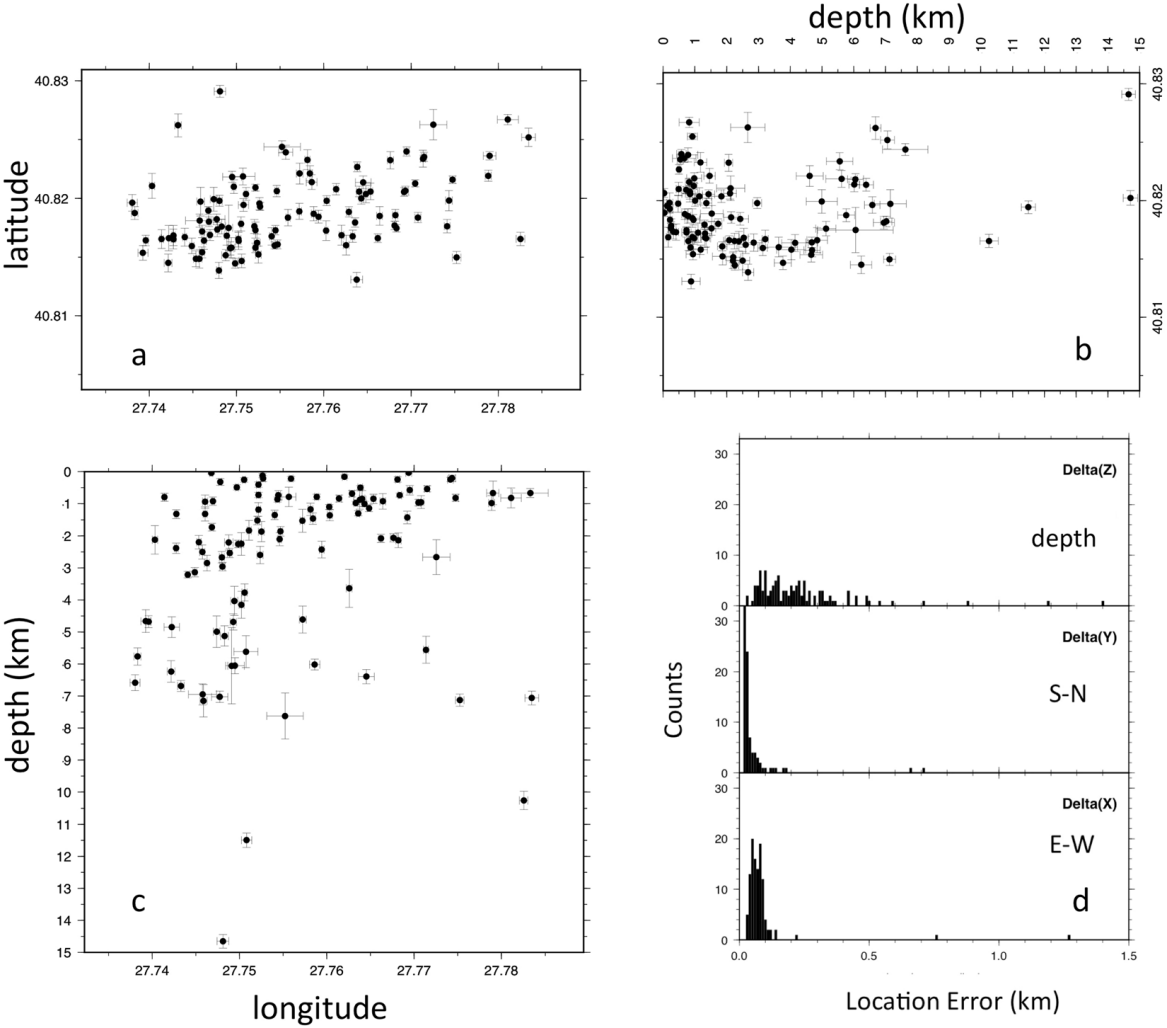
Location errors of the aftershocks shown in Fig. 2. (**a**) latitude-longitude plane; (**b**) longitude-depth plane; (**c**) latitude-depth plane. Panel d) displays the distribution of location errors in the 3 directions: depth (deltaz); South-North (delta (y)); Est-West (delta(x)). Image created with GMT software, Version 4.5.11.

The results presented here (Fig. 2) were obtained using a 3D-model that was specifically tailored for the 20 km × 60 km area covered by the offshore network, with a grid node spacing of 750 m × 750 m × 200 m, using all available geological and geophysical information for the Sea of Marmara (see ref.^25^ and details in Appendix 1). Different location methods were tested^32,33^. Here we present results obtained using the NLLOC inversion algorithm^32,33^ for absolute location, and NLDiffLOC^33,34^ for relative locations, which are less sensitive to the event location and origin time. This turned out to be possible only over a limited number of events (112 in total), due to computation instabilities, which resulted in “water” relocations for the most shallow events^35^. The resulting location errors (Fig. 3) are less than about 200 m on the horizontal components (E-W and N-S). In contrast, on the vertical component, depth location errors are distributed between 0 and 500 m for most events (only 7 events out of 112 have an error >500 m); as expected, errors (expressed as the ratio between error and depth) dramatically increase for the most shallow earthquakes.

**Figure 4:**
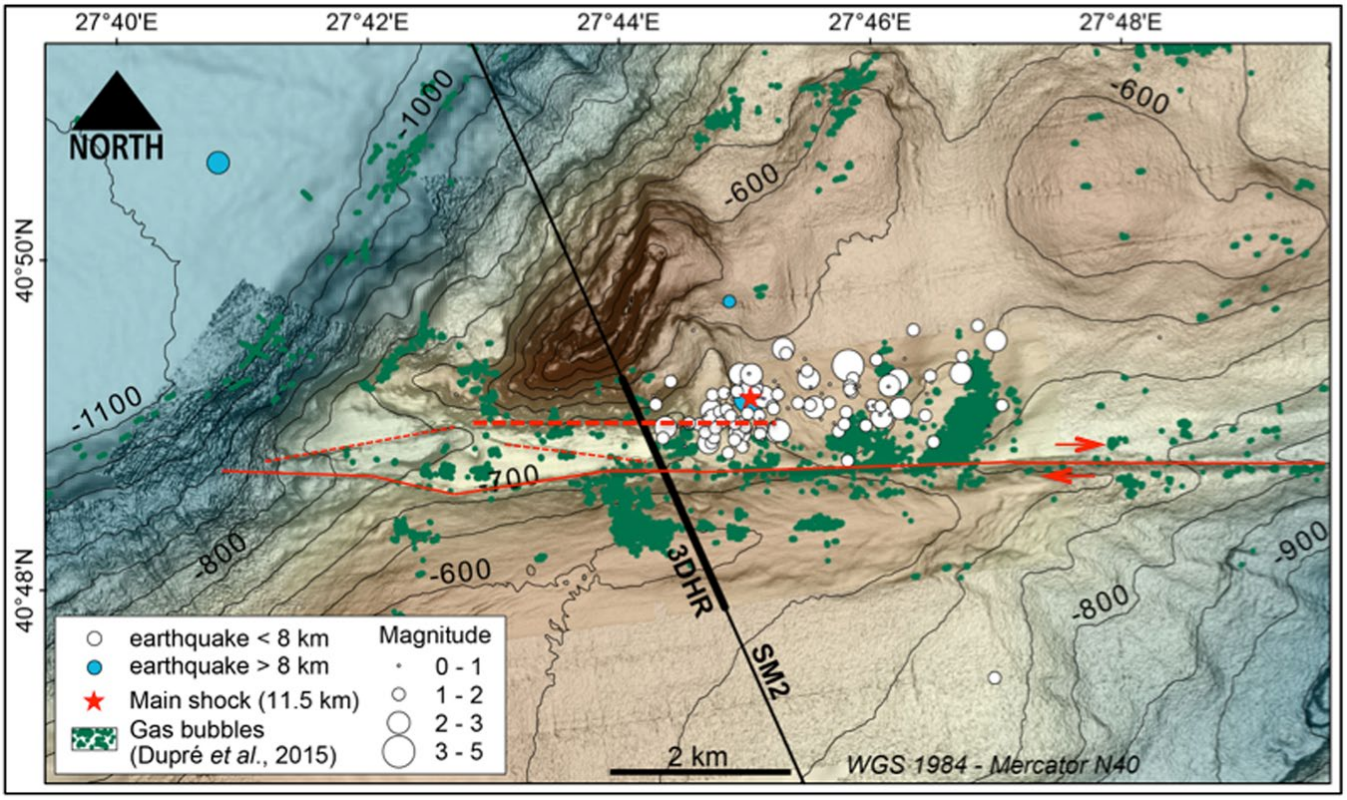
Epicenter locations for the aftershocks that followed the M 5.1 earthquake of July 25, 2011, superposed on a high-resolution bathymetric grid derived from a combination of near-seafloor AUV data^37^ and on an unpublished, bathymetric grid having a resolution of 10 m, based on data collected in 2014 during the Marsite cruise of R/V Pourquoi pas?. White and blue dots are respectively epicenters within and below the sedimentary basin. Green dots indicate the location of the gas emission that were acoustically detected^22^ in 2009, using the multibeam echasounder EM302 of R/V Le Suroit. Note that there is no clear overlap between the seismicity (from 2011) and seafloor gas emissions (detected in 2009), e.g. there is no direct evidence that the ultra-shallow aftershocks of 2011 directly induced gas emissions in the water column. Thin black line indicates the track of the seismic section displayed in Fig. 5. The thickened segment represents the location of the 3D, High-Resolution seismic image shown in Fig. 6. The continuous red line indicates the trace of the right-lateral, strike-slip, Main Marmara Fault. Red dotted lines show secondary faults indicating the complexity of the fault network linked to the MMF. The thicker, east-west oriented, dotted line is also shown in the seismic section displayed in Fig. 5. Image created with Arc-GIS, version v3.0.

Only a few events are located within the crystalline basement, at crustal depths greater than 8 km. Most aftershocks are located within the 6 to 8 km thick sedimentary pile that forms the Western High^31^, with a great number of them located within the uppermost layers, at depths shallower than ~1.5 km. The zoomed view (Fig. 4) reveals that the epicentres of the mainshock and of all aftershocks are located to the north of the fault trace. Within the Plio-Pleistocene sedimentary pile, aftershocks are not all located along the main fault plane. In addition, the mainshock is not exactly located at the apex of the surface trace of the Main Marmara Fault, but ~ 800 m to the north, which suggests that the main fault plane may not be not strictly vertical, but slightly inclined (by about 4°) relatively to the vertical.

**Figure 5:**
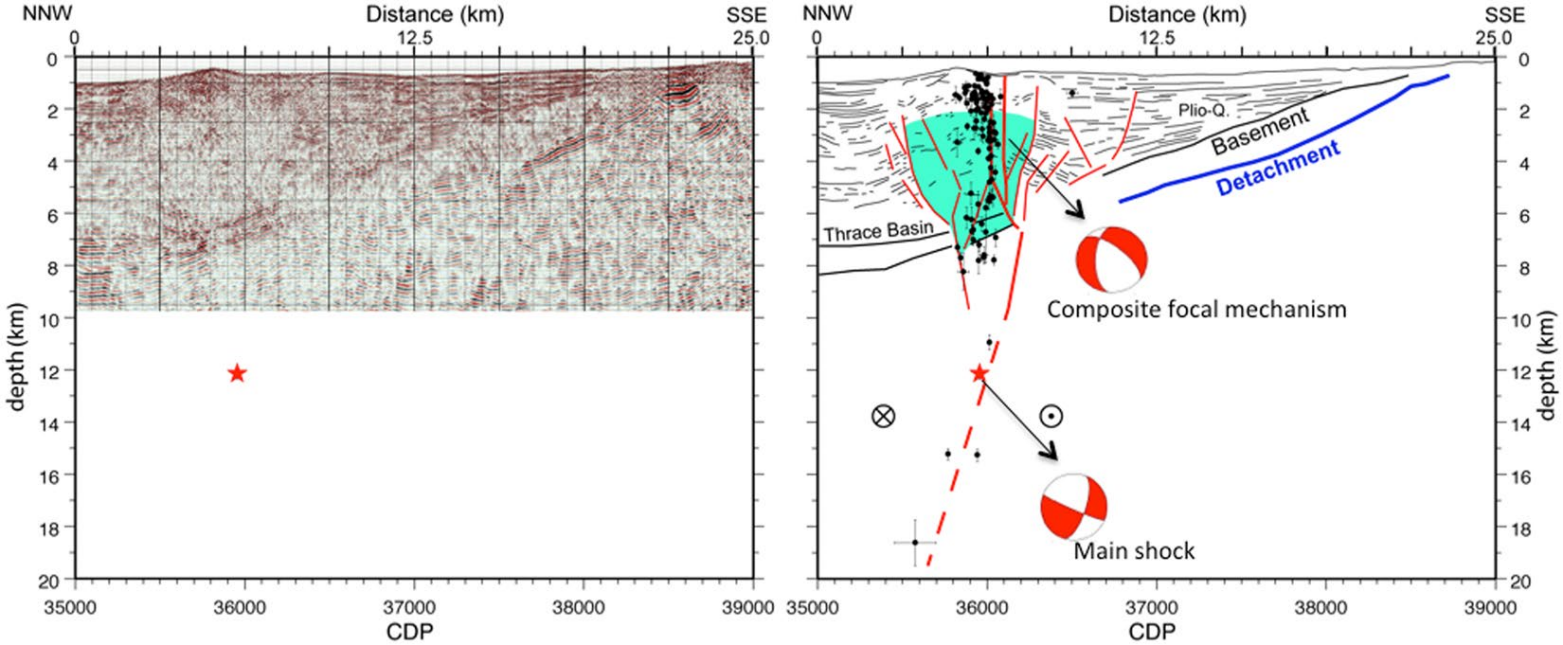
Left panel displays the hypocenters of the aftershock sequence, superimposed on the seismic section (see track line in Fig. 2) that was shot during the Seismamara cruise^28,36,56^ in 2001 in the Western High area. The migration was obtained after stack and time-migrated, by using 5 main velocity intervals based on velocity analysis for the sedimentary layers and constraints from refraction modelling for the crust: the water-column, the shallow sediment interval, the deep sediment interval, the interval between the basement and the detachment, and the crustal layer below the detachment. Velocity in the water is settled constant at 1500 m/s and velocity increases linearly in other intervals. The average velocity in the shallow sediment is 1700 m/s, 2400 m/s for the deep sediment interval, 3300 m/s between the acoustic basement and the detachment, and is 5500 m/s in the crust below the detachment. Line-drawing (partly based on ref.^37^, page 160) is displayed in right panel along with the locations of the aftershocks that followed the M 5.1 earthquake of July, 25, 2011. Continuous red line indicates the MMF. The dotted line within the sedimentary basin indicates the east-west oriented, secondary fault displayed in Fig. 4. The earthquakes (at depths between 2 and 4 km) that were used to compute the composite focal mechanism using HASH^38,39^ are listed in Appendix 3. Image created with GMT software, Version 4.5.11.

The aftershocks were superimposed on the multichannel seismic section collected in 2001, during the Seismarmara cruise^28,36^ (see also in ref.^37^, page 160). Different groups of aftershocks can be described, depending on depth (Fig. 5):

i between 5 and 7 km below seafloor (bsf), aftershocks appear to occur at the base of the pre-kinematic basement, along the main fault but also along secondary faults that are known to intersect the Main Marmara Fault, based on deep, seismic imaging;
ii between ~5 and 1.5 km bsf, aftershocks appear to be aligned along a secondary fault, related to the on-going opening of a small pull-apart basin on the western side of the Western High. The composite focal mechanism computed with HASH^38,39^ for a number of well defined earthquakes (see list in Appendix 3) indicate predominant normal faulting;
iii at depths above 1.5 km, where a great number of aftershocks occurs, the epicentres are spread out away from the fault trace, within gas-prone sedimentary environments.

To examine the potential role of gas, let us now provide details on the gas that were sampled in the Western High area. Geochemical analysis^23^ have shown that the gases are of thermogenic origin, with a composition similar to Eocene Thrace Basin gas fields^40^. However, the isotopic composition of gases emitted at the seafloor indicate oil biodegradation and secondary generation of microbial methane at less than 80 °C^41,42^, that may have occurred within a reservoir or during migration. The pore fluids sampled from sediment strata at the same site where liquid hydrocarbons and gas are expelled are enriched in chloride, lithium, strontium and barium^43^. The application of different geo-thermometers indicates fluid/sediment interaction within the temperature window of ~75 to 150 °C, a narrower 75–130 °C range being obtained with Li geo-thermometers, commonly applied to oil-bearing sedimentary basins^42^. The expulsion of brines together with oil and gas suggests that these seepage sites are fed by leakage from an over-pressured zone, which is also supported by the diapiric nature of the fluid conduit (see Supp. Mat., Appendix 5). The heat flow measured at the surface is lowered by the effect of sediment blanketing, with a mean value of 35 ± 7 mW/m2, however basin models^44^ constrain the probable range of crustal heat flow below the western high between 50 and 70 mW/m2. Temperature vs. depth profiles were calculated using different models to describe the evolution of conductivity with depth^45^ and assuming that the heat transfer at the basin scale is conductive (advection is neglected) (see details in Supp. Mat. Appendix 4). Within all uncertainties, the possible depth range of the source of over-pressured fluids is thus estimated as 2-to-5-km depth. It remains possible that gas and pressure generation also occurs at greater depths where Thrace Basin source rocks are present as thermogenic gas generation can occur up to more than 200 °C.

**Figure 6:**
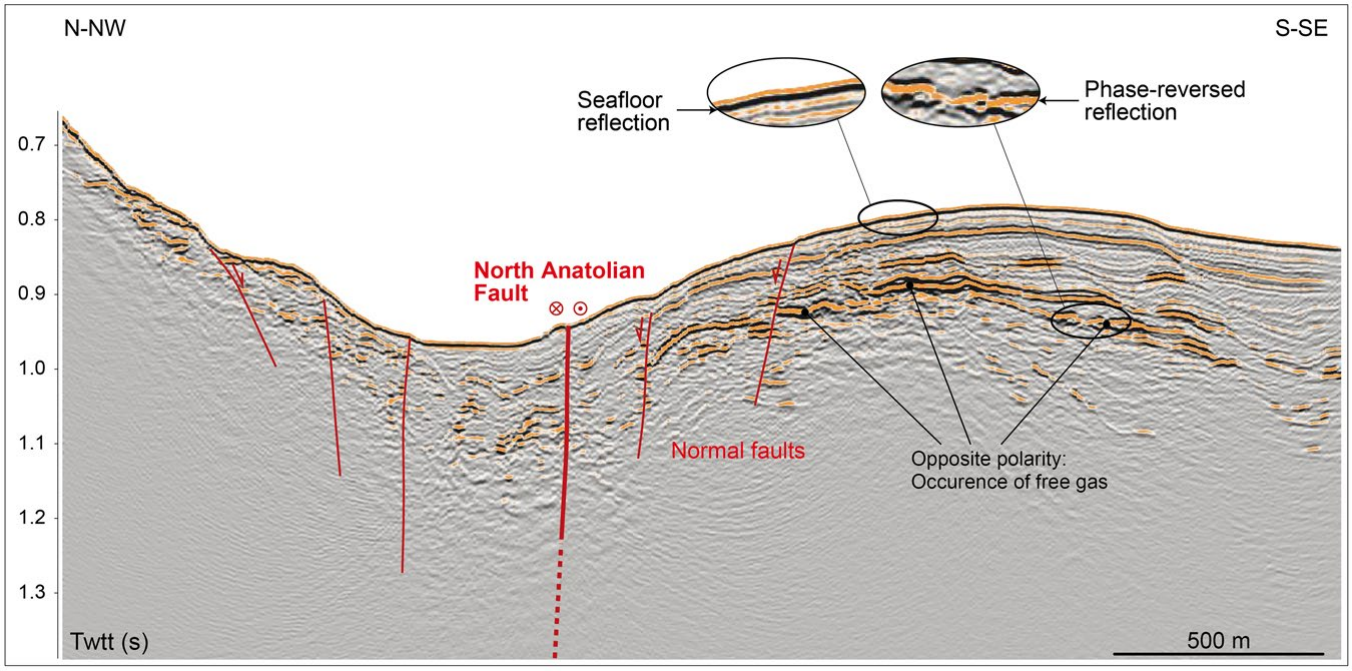
Seismic section extracted from the high-resolution 3D seismic box shot in 2009^46^ along the thick black line displayed in Fig. 5, with a simplified, tentative interpretation. Image created with Arc-GIS, version v3.0.

Many sources could be on the way from the source rock to the seafloor, with different geochemical or biogeochemical processes occurring at different levels. At shallow levels, the gas migration pathways were mapped down to a few hundred meters beneath the Western High seabed, using 3D, high-resolution seismic data collected in 2009^46^ (Fig. 6). All over the 3D survey area, reflections of very strong amplitude and opposite seismic polarity compared to that of the surrounding seabed suggest the presence of free gas immediately below the reflecting horizons. This view is coherent with both acoustic, offshore surveys and visual observations showing that, where horizons are faulted and/or crop out at the seafloor, gas emissions are observed in the water column. In contrast, at unfaulted locations, several horizons appear to collect the gas migrating from depth. This is probably because they are of higher porosity and permeability than the dominant, clay-rich lithology. The data also reveal that gas follows buoyancy-driven, upward migration paths in permeable layers and along faults, controlled by the regional strain field as it is expressed in the seafloor topography, with the primary E-W orientation parallel to the NAF and secondary tectonic orientations oblique to the NAF^47^. Locally, mud volcano-like structures may also offer preferential pathways for the gas to migrate up to the seafloor.

The general picture that finally comes out is that gas is conveyed from gas-rich, deep sources (located between ~1.5 and ~5 km) up to the seafloor along the Main Marmara fault zone system. Beneath the main fault valley, gas tends to follow buoyancy-driven migration paths through permeable layers, up to the crest of the anticline folds that border the main fault valley (Supp. Info., Appendix 5). This suggests that most of the aftershocks that occurred within and above the gas window are likely linked to gas-related processes, e.g. the mainshock triggered not only “purely tectonic”, but also “gas-related” aftershocks. The main shock could have triggered the pressurization and activation of the faults located within or above the deepest gas sources. Alternatively, gas ex-solution from sediment pore fluids could have occurred in response to the main shock; the resulting increase of compressibility of the fault material could have then triggered some of the aftershocks, as predicted by the Piau-Maury-Fitzenz model^48^, which was successfully used to describe the effect of fluid compressibility on the rupture process at oceanic fracture zones^49^. It is important to note that soft, marine sediments (with velocities < 2 to 3 km/s^13,35^) are generally not expected to be able to host earthquakes. However, recent results from laboratory experiments on clays at high slip-rates (i.e. >2 m/s)^50,51^ have shown that clays can react seismically to rupture that propagates into it, one such example being the 2011 Tohoku earthquake where 30 m of slip is estimated to have occurred in clay like material^52^. A similar scenario could apply to explain the ultra-shallow events that occurred at depths of less than a few hundred meters. Also, it is likely that the gas-prone sediment layers that were present in 2009, based on high-resolution 3D seismics, were still there in 2011 and that the aftershocks were triggered by gas over-pressurization. In contrast, because the 2009 cruise predates the aftershock sequence of 2011, there is no direct evidence that the ultra-shallow aftershocks of 2011 directly induced new gas emissions in the water column.

In conclusion, the present work reveals the existence of shallow micro-seismicity in the Sea of Marmara. Most events of the aftershock sequence appear to be located within or above the gas window below the Western High, at shallow (2–5 km) and ultra-shallow (<2 km) levels. It is hence suggested that part of these aftershocks are likely gas-induced. The characterization of micro-seismicity as evidence -or not- of creep along the North Anatolian fault segments^8,53^, as well as the search for seismic tremors, similar to those that occurred prior to the 1999, Izmit earthquake^54^, are challenges of critical importance for seismic hazard assessment and mitigation within the “Istanbul seismic gap”. However, the precise hypocenter location and the classification of micro-seismicity into either tectonic earthquakes, either gas-related events, are prerequisites. These can be achieved only by the implementation of permanent, networks of deep seafloor observatories in the immediate vicinity of the fault.

## Methods

### Building a high-resolution, 3D-velocity model (see Figures in Supp. Mat., Appendix A1)

A model with a 750 m × 750 m × 200 m grid spacing was built, for the Western Sea of Marmara (40°43′N–40°54′N–27°30′E–28°15′E), in order to account for the velocity contrast at the water/sediment interface and for the slow seismic velocities within the sediment infill in the main Marmara Trough. All available, multibeam bathymetry and wide-angle seismic data from the area were used. The model was developed following the six steps described below^25^:

1. The tomographic model of *Bayrakci et al*.^31^ was used to describe the velocity structure of the pre-kinematic basement and the velocity structure down to 12 km below the Marmara sea-level. This model (see Figure 13a in ref.^31^) is based on a low-resolution grid of 6 km × 6 km × 2 km. The iso-velocity contours of the pre-kinematic basement were superposed to the bathymetric map and used as guide lines to define 9 “basement domains” (Figs A1–1).
2. For each domain, a “typical” velocity profile down to 12 km depth was calculated by averaging all velocity profiles within the given domain (Figs A1–2a, A1–2b, A1-2c).
3. A dense, high-resolution sub-grid was then defined (Figs A1–3), with grid spacing 750 m × 750 m × 200 m, by sub-dividing the tomographic grid of *Bayrakci et al*.^31^.
4. Each node M of the dense sub-grid was ascribed: i) to the water depth inferred from the high resolution bathymetric grid of *Le Pichon et al*.^6^; ii) to a given domain N (with N = 1 to 9, as defined in Figs A1–3). The velocity structure at grid node M for the upper 12 km is provided by the characteristic velocity profile of domain N.
5. Below 12 km and down to 36 km, the velocity structure is assumed to depend on longitude and inferred from wide-angle reflexion results (see Fig. 3 of *Bécel et al*.^55^. Velocities of 6.7 km/s and 8 km/s were ascribed to the lower crust and upper mantle respectively (see example in Figs A1–4).
6. Each point of the fine sub-grid is thus characterized by: the exact depth at grid node, the “domain” number, the “typical” velocity profile above 12 km, the depth of lower crust and the depth of Moho.

The high-resolution grid was used for computing absolute and relative locations using Lomax’s software^33^. For computing relative locations using HypoDD-3D^32^, a degraded, 3D grid was used due to grid size limitations and to avoid border effects.

### Geotherm estimation below the Western High (see Figures in Supp. Mat., Appendix A4)

To estimate the depth range at which temperatures ranging between 75 °C and 80 °C might be expected, eight thermal profiles (e.g. sediment temperature versus depth below seafloor) were obtained (see plots in Figs A4–2) along with thermal conductivity measurements performed on co-located cores. The observed spatial variability of thermal gradients (Figs A4–2) suggests that the heat transfer to the surface is likely influenced by a variety of processes (including sediment thermal blanketing, fluid circulation, gas hydrate related perturbation, topography, etc) that appear difficult to model, mainly due to the scarcity of thermal measurements. We therefore rather use the initial heat flow value of 68.10^−3^ W.m^−2^ that was derived by *Grall et al*.^47^ from the detailed study of the thermal and subsidence history of the Central Basin. Indeed, the sedimentary column is thick at the Western High but sedimentation rate since at least the last hundred thousand years is no greater than 1.5 mm/a^47^. Thus the sediment thermal blanketing should not change drastically the present-day heat flow at the seafloor.

Let us consider that: (i) that this value (hereafter referred to as Q_b_), represents a reasonable proxy for the basal heat flow below the Western High area; and (ii) that at the scale of the area, heat flow is conductive, e.g. conservative.

Then:

1 $$k(z)\frac{dT}{dz}={Q}_{b}$$

which yields:

2 $$T(z)={T}_{0}+{\int }_{0}^{z}\frac{{Q}_{b}}{k(z)}dz$$

where T(z) and k(z) are temperature and thermal conductivity, respectively. Following *[Pribnow et al*., *2000]*^45^, we have tested two different approaches to describe the variation for porosity with depth:

- The linear approach:
  3 $$k(z)={k}_{0}+Az$$
  4 $$T(z)={T}_{0}+\frac{{Q}_{b}}{A}Log(1+\frac{Az}{{k}_{0}})$$
  where k_0_ and A are the thermal conductivity at the surface (0.83 W K^−1^ m^−1^) and the thermal conductivity gradient, respectively. Using compilations^45^, based on the data collected during Legs 101 to 180 of the Ocean Drilling Programme, we tested different values for A (from 0.4 to 1.4 × 10^–3^ W K^−1^ m^−2^).
- The “porosity approach”, which assumes that k(z) depends on porosity and that porosity exponentially increases with depth due to compaction:

5 $$\phi (z)={\phi }_{0}{e}^{-az}$$

and

6 $$k(z)=\phi (z){k}_{w}+(1-\phi (z)){k}_{g}$$

7 $${k}_{g}={k}_{0}+\frac{{\phi }_{0}}{1-{\phi }_{0}}({k}_{0}-{k}_{w})$$

where φ(z) and φ_0_ are porosity at depth z and at sediment surface, respectively, while a stands for Athy’s compaction factor, k_w_ and k_g_ for thermal conductivity of seawater and sediment grains. The thermal conductivity of grains (k_g_) is derived from surface sediment porosity and conductivity as stated in (7).

Using the above formulae, an analytical expression of temperature is found for integral in (1):

8 $${\rm{T}}({\rm{z}})={{\rm{T}}}_{0}+\frac{{Q}_{b}}{{k}_{g}a}Log|\frac{k(Z)}{{k}_{0}}\frac{{\phi }_{0}}{\phi (z)}|$$

Using bottom water temperature of 14 °C, temperatures at depth z are found, based on expressions (4) or (8).

## Electronic supplementary material

Supplemtary information
