# Supplementary material for: Gas and seismicity within the Istanbul seismic gap
Source: Sci Rep. 2018 May 1;8:6819. doi: 10.1038/s41598-018-23536-7 (PMC5931589; doi:10.1038/s41598-018-23536-7)
Supplement: Supplementary file 1 — Supplemtary information [file 41598_2018_23536_MOESM1_ESM.pdf]

SUPPLEMENTARY INFORMATION

Manuscript title: Gas and seismicity within the Istanbul seismic gap

Authors list: L. Géli[1], P. Henry[2], C. Grall[2,3], J.-B. Tary[1,4], A. Lomax[5], E. Batsi[1], V. Riboulot[1], E., Cros[1], C. Gürbüz[6], S. Isik[6], A. M. C. Sengör[7], X. Le Pichon[2], L. Ruffine[1], S. Dupré[1], Y. Thomas[1], D. Kalafat[6], G. Bayrakci[1,8], Q. Coutellier[1], T. Regnier[1], G. Westbrook[1,9], H. Saritas[10,11], G. Cifçi[11], M. N. Çağatay[7], M. S. Özeren[7], N. Görür[7], M. Tryon[8], M. Bohnoff[12], L. Gasperini[13], F. Klingelhoefer[1], C. Scalabrin[1], J.-M. Augustin[1], D. Embriaco[14], G. Marinaro[14], F. Frugoni[14], S. Monna[14], G. Etiope[14], P. Favali[14], A. Bécel[3]

[1]Ifremer, Centre de Bretagne, Plouzané, France
[2]CEREGE, Aix-Marseille University, CNRS, Marseille, France
[3]Lamont-Doherty Earth Observatory, Palisades, NY, USA
[4]Universidad de los Andes, Bogotà, Colombia
[5]ALomax Scientific, 06370 Mouans-Sartoux, France
[6]Kandilli Observatory and Earthquake Research Institute, Boğaziçi University, Istanbul, Turkey
[7]*Istanbul Technical University, Istanbul, Turkey*
[8]Ocean and Earth Science, National Oceanography Centre, Southampton, UK
[9]School of Geography, Earth and Environmental Sciences, University of Birmingham, UK
[10]Mineral Research & Exploration General Directorate, MTA, Ankara, Turkey
[11]Institute for Marine Science and Technology, Dokuz Eyiul Universitesi, Izmir, Turkey
[12]Deutsches Geo-Forschungs Zentrum, GFZ, Potsdam, Germany
[13]Institute of Marine Science, ISMAR-CNR, Bologna, Italia
[14]INGV, Roma, Italia

APPENDIX 1: Building a specifically designed, high-resolution, 3D, seismic velocity model for the Western Sea of Marmara

Written by Louis Géli, Quentin Coutellier, Jean-Baptiste Tary, Gaye Bayrakci
& Cemil Gürbüz

In parallel to the study reported here, a complementary study was conducted within the EU-Funded MARSITE Project (www.marsite.eu), with among other targets, the objective to improve the characterization of the near-fault micro-seismicity, particularly along the central part of the SoM. To meet this objective, velocity models were developed by KOERI and by Ifremer to improve earthquake location in the Sea of Marmara, following two different approaches:

- KOERI has developed a 3D velocity model for the whole Marmara Region (within latitudes 39.5°N - 42.5°N and longitudes 26.0°E - 30.5°E), including land and seabottom stations, with grid spacing of 9 km x 9 km x 3 km (*Gürbüz et al, [2013], Isik [2014]*).

- Ifremer has developed a different, but complementary approach, for the Western Sea of Marmara (40°43'N - 40°54'N – 27°30'E – 28°15'E). A high resolution velocity model with a 750 m x 750 m x 200 m grid spacing was built, using multibeam bathymetry and wide-angle seismic data, in order to account for the velocity contrast at the water/sediment interface and for the slow seismic velocities within the sediment infill in the main Marmara Trough (*Cros and Géli, [2013]*).

The fine-scale 3D-velocity model was developed following the six steps described below:

1. The tomographic model of *Bayrakci et al [2013]* was used to describe the velocity structure of the pre-kinematic basement and the velocity structure down to 12 km below the Marmara sea-level. This model (see Figure 13a of Bayrakci *et al*, [2013]) is based on a low-resolution grid of 6 km x 6 km x 2 km. The iso-velocity contours of the pre-kinematic basement were superposed to the bathymetric map and used as guide lines to define 9 domains (Figure A1-1).
2. For each domain, a "typical" velocity profile down to 12 km depth was calculated by averaging all velocity profiles within the given domain (Figures A1-2a, A1-2b, A1-2c).
3. A dense, high-resolution sub-grid was then defined (Figure A1-3), with grid spacing 750 m x 750 m x 200 m, by sub-dividing the tomographic grid of *Bayrakci et al. [2013]*.
4. Each node M of the dense sub-grid was ascribed: i) to the water depth inferred from the high resolution bathymetric grid of *Le Pichon et al. [2001]*; ii) to a given domain N (with N=1 to 9, as defined in Figure A1-3). The velocity structure at grid node M for the upper 12 km is provided by the characteristic velocity profile of domain N.
5. Below 12 km and down to 36 km, the velocity structure is assumed to depend on longitude and inferred from wide-angle reflexion results (see Figure 10 of *Bécel et al. [2009]*. Velocities of 6.7 km/s and 8 km/s were ascribed to the lower crust and upper mantle respectively (see example in Figure A1-4).
6. Each point of the fine sub-grid is thus characterized by: the exact depth at grid node, the "domain" number, the "typical" velocity profile above 12 km, the depth of lower crust and the depth of Moho.

The high-resolution grid was used for computing absolute and relative locations using Lomax's software (e.g. *[Lomax et al, 2000], [Lomax et al, 2009]*). For computing relative locations using HypoDD-3D *[Waldhauser, 2001*], a degraded, 3D grid was used due to grid size limitations and to avoid border effects.

References:

Bayrakci G., Laigle, M., Bécel, A., Hirn, A., Taymaz, T., (2013), 3-D sediment-basement tomography of the NorthernMarmara trough by a dense OBS network at the nodes of a grid of controlled source profiles along the North Anatolian fault, *Geophys. J. Int*., doi: 10.1093/gji/ggt211

Bécel, A.; Laigle, M.; de Voogd, B.; Hirn, A.; Taymaz, T.; Galvé, A.; Shimamura, H.; Murai, Y.; Lépine, J.-C.; Sapin, M. & Özalaybey, S. (2009). Moho, crustal architecture and deep deformation under the North Marmara Trough, from the SEISMARMARA Leg 1 offshore-onshore reflection-refraction survey, *Tectonophysics* **467**: 1-21

Cros, E., & Géli, L. (2013), Caracterisation of microseimicity in the Western Sea of Marmara: implications in terms of seismic monitoring, *Project Report, Institut Carnot Ifremer-Edrome, Abondement 2011, N°06/11/2013, 29 pages,* available on-line: http://dx.doi.org/10.13155/38916

Gürbüz C., Isik S. E., Geli L., Cros E. (2013). High Resolution Micro Earthquake Characterization, *Deliverable D8_2, EU MARSITE PROJECT (New Directions in Seismic Hazard Assessment through Focused Earth Observation in the Marmara Supersite)*. http://archimer.ifremer.fr/doc/00278/38915/

Işık, S. E. (2014) 3D, P-wave velocity structure of the Marmara Region using local earthquake tomography, *Masters Thesis*, Bogazdici Universitesi.

Le Pichon, X., A. M. C. Sengor, E. Demirbag, C. Rangin, C. Imren, R. Armijo, N. Gorur, and N. Cagatay (2001), The active Main Marmara Fault, *Earth Planet. Sci. Lett.,* **192**, 595–616.

Lomax, A., A. Michelini, A. Curtis (2009), Earthquake Location, Direct, Global-Search Methods, in Complexity In *Encyclopedia of Complexity and System Science, Part 5, Springer, New York,* pp. 2449-2473, doi:10.1007/978-0-387-30440-3.

Lomax, A., J. Virieux, P. Volant and C. Berge, (2000), Probabilistic earthquake location in 3D and layered models: Introduction of a Metropolis-Gibbs method and comparison with linear locations, in *Advances in Seismic Event Location*, Thurber, C.H., and N. Rabinowitz (eds.), Kluwer, Amsterdam, 101-134.

Waldhauser, F., HypoDD: A computer program to compute double-difference earthquake locations, *USGS Open File Rep.,* **01-113**, 2001. pdf

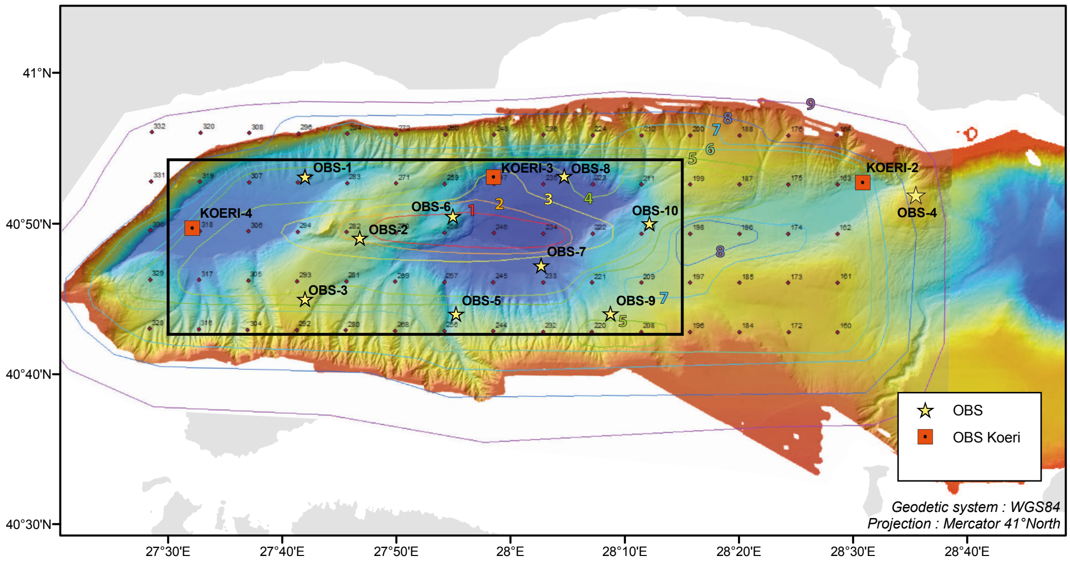

Figure A1-1: Bathymetric of the Western Sea of Marmara, based on the high-resolution, 38-m grid from Le Pichon et al, [2001], with iso-contours of pre-kinematic basement depth, from Figure 13a of Bayrakci et al [2013]. Iso-contours define 9 domains (labelled from 1 to 9), characterized by a specific velocity profile. Numbered black dots are nodes of the low-resolution grid (6 km x 6 km x 2 km) of Bayrakci [2013]. Black, rectangle frame indicates the area selected for the definition of the high-resolution grid shown in Figure A1-3. Yellow stars are Ifremer's autonomous, short period OBSs. Red squares are KOERI's permanent, cabled OBSs. Image created with GMT software, Version 4.5.11.

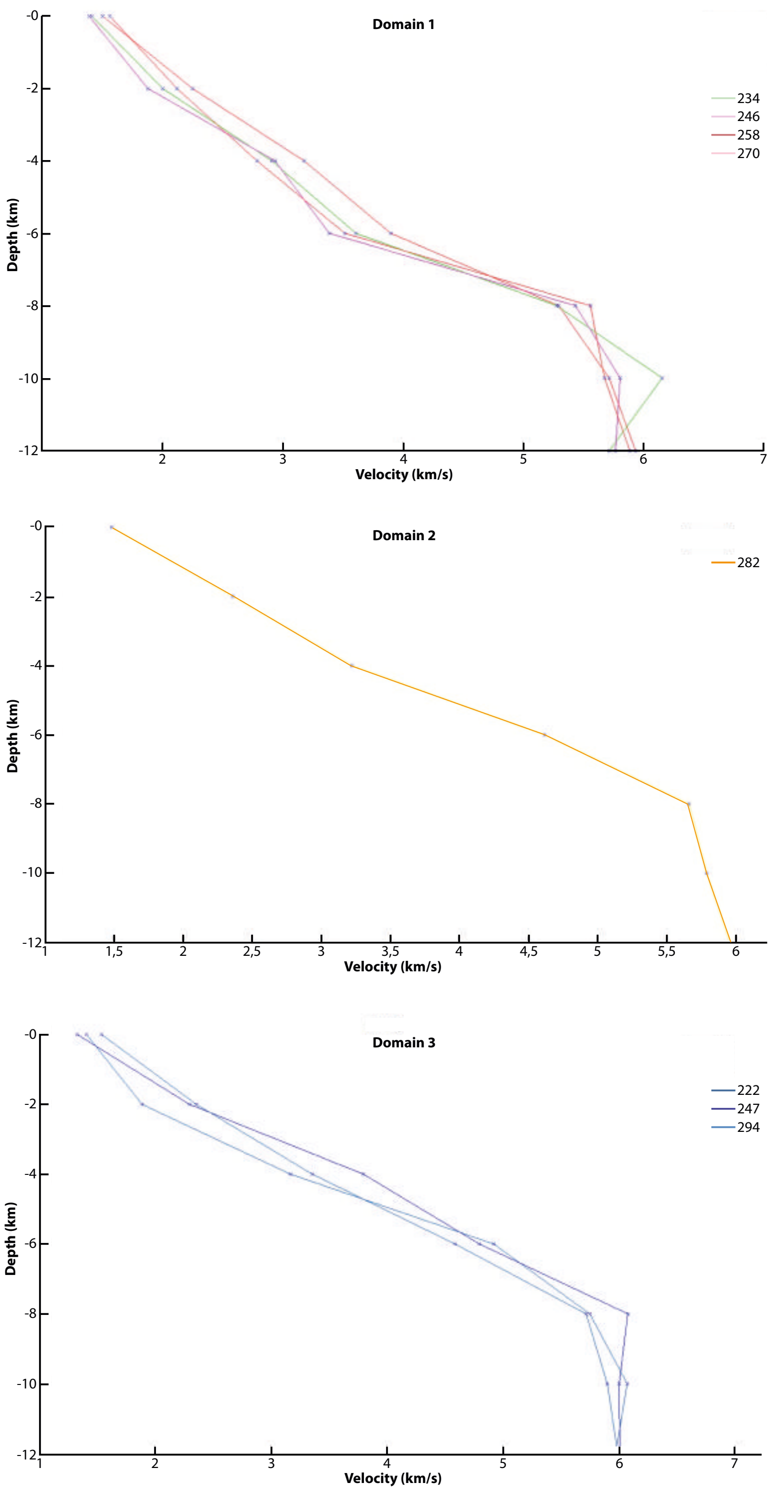

Figure A1-2a: Vertical velocity profiles (from 0 to 12 km depth below seafloor) below each node of the low-resolution, tomographic grid of Bayrakci et al [2013], within, respectively domains 1, 2 and 3 (see domain delineation in Figure A1-1). Image created with GMT software, Version 4.5.11.

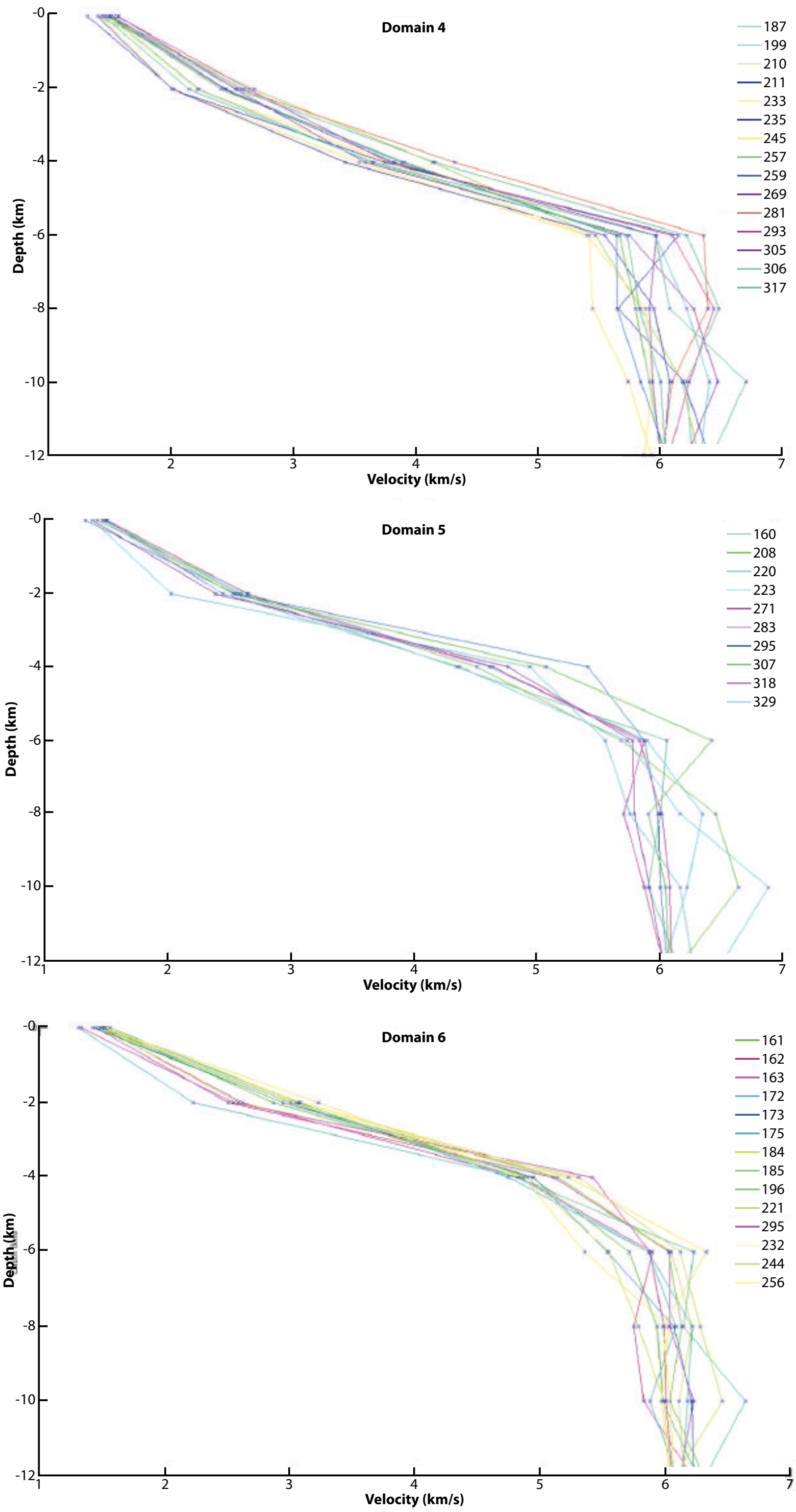

Figure A1-2b: Vertical velocity profiles (from 0 to 12 km depth below seafloor) below each node of the low-resolution, tomographic grid of Bayrakci et al [2013], within, respectively domains 4, 5 and 6 (see domain delineation in Figure A1-1). Image created with GMT software, Version 4.5.11.

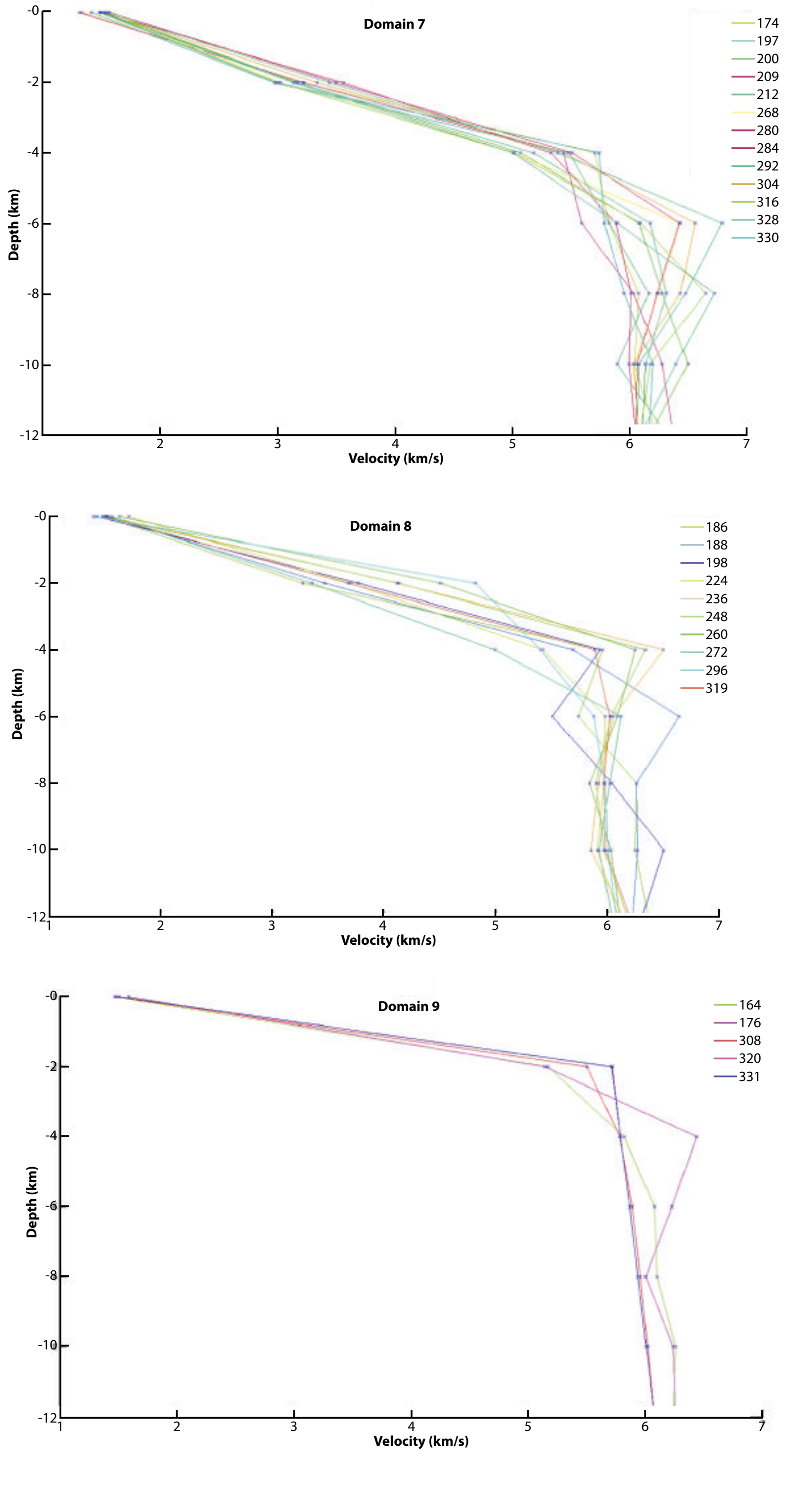

Figure A1-2c: Vertical velocity profiles (from 0 to 12 km depth below seafloor) below each node of the low-resolution, tomographic grid of Bayrakci et al [2013], within, respectively domains 7, 8 and 9 (see domain delineation in Figure A1-1). Image created with GMT software, Version 4.5.11.

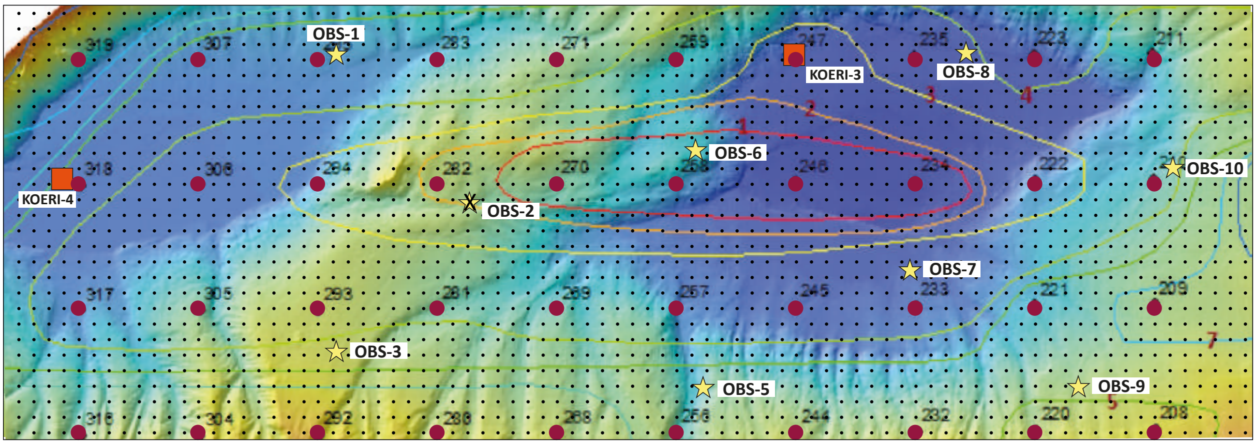

Figure A1-3: Bathymetric map of study area within the Western Sea of Marmara. Black dots indicate node locations for the high-resolution grid, with spacing of 750 m x 750 m x 200 m. Red dots indicate node locations for the low-resolution (6 km x 6 km) tomographic grid of *Bayrakci et al. [2013*]. Labels from 1 to 9 on basement iso-contours indicate domain number (see caption Fig. A1-1). KOERI's cabled OBSs and Ifremer autonomous OBSs are marked as red squares and yellow stars, respectively. The yellow star corresponding to Ifremer OBS-2 is crossed because OBS-2 stopped recording before the M_l 5.1 earthquake of July 25th, 2011. *Image created with ArcGIS, v3.*

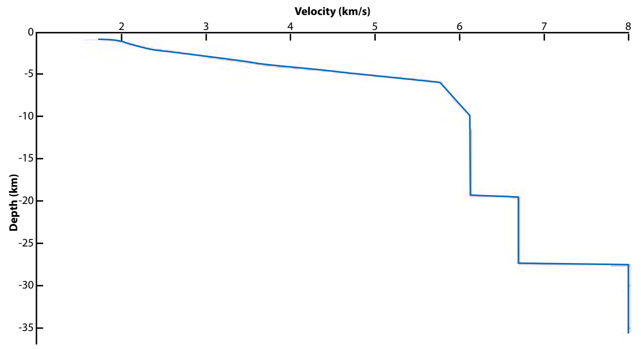

Figure A1-4: Example of velocity profile finally obtained for node located within domain 4, at longitude 27°37.56'E, latitude 40°46.50'N. The typical velocity profile of domain 4 is used for z < 12 km. For z > 12 km, velocities and interface depth for lower Crust and Moho depth are inferred as a function of longitude, based on wide-angle and refraction seismics results (e.g. see Figure 10 of Bécel et al [2009]). Image created with GMT software, Version 4.5.11.

APPENDIX 2

Testing velocity models on aftershocks relocation

Written by Estelle Cros
With contributions of Louis Géli, Anthony Lomax and Jean-Baptiste Tary

The aftershock sequence following the M_w 5.1 earthquake of July, 25th, 2011

To monitor the fault segment of the NAF in the SoM, a non-permanent network of ten 0BSs with four components was deployed (Figure A2-1) and recorded data during 107 days, from April 15th to July 31st (unfortunately OBS 2 failed and stopped recording after June 30th, 2011). The network was designed to complement the permanent submarine monitoring system of the Kandilli Observatory and Earthquake Research Institute (KOERI) and to carry out a high resolution characterization of earthquakes within the Western part of the SoM, including the Tekirdag basin, the Western High and the Central basin *[Cros and Géli, 2013].*

KOERI's cabled OBSs were permanent stations, equipped with GURALP CMG-3, broad-band seismometers, synchronized with GPS clocks. In contrast, Ifremer OBSs were autonomous (non-permanent) equipped with 3-component, short-period (4.5 Hz) seismometers and one hydrophone. *In-situ* photographs have shown that seismometer capsules are entirely covered by sediments, providing reasonable coupling with the seafloor [*e.g. Tary et al, 2011*]. Based on laboratory results obtained in comparable pressure and temperature, the drifts of the OBSs' internal clocks (~0.3 s per month) were linearly corrected for each instrument.

Event detection was first performed using a STA/LTA algorithm through the Sytmis® software. The problem found was that electronic signals and non-seismic, short-duration events (e.g. *[Tary et al, 2012]*, *[Embriaco et al, 2013]*) were also detected by the algorithm. Hence, the picking of the first arrivals was done manually and all these type of events were removed during this process. Almost 700 events were detected and picked manually (Figure A2-2). Most of the seismicity occurred during July 2011, with an amount of ~550 events. This high rate of seismicity is associated to a sequence of aftershocks that followed a Mw 5.2 earthquake, which occurred on July 25th at 17h57 (Foigure A2-3).

Manual picking on the OBS network proved to be very efficient to improve the detection threshold. For comparison: the catalog available on the website of the European-Mediterranean Seismological Center (EMSC) reports 145 events during the recording period, with only 70 in july 2011 (Figure A2-2). An automatic method for picking, earthquake association and location was also developed by *Lomax [2014]*. This automated method proved to be also efficient, as ~380 aftershocks were eventually located. Hereafter, we only report the results obtained from manual picking.

Velocity models

Earthquake location was performed step by step, using 1D- and 3D- velocity models. Four, 1D-models were compared (Figure 4):

- the model of *Tary et al. [2011]*
- a model based on *Bécel [2006]* and *Bécel et al [2009]*
- the model of *Gurbuz et al. [2000]*,
- an average velocity model based on all these previous models.

The model derived by *Gurbuz et al. [2000]* is well adapted for on shore network, but does not account for the effect of the slow-velocity, surficial sediment layers. The model of *Bécel et al [2006]* is derived from wide-angle seismic data from an East-West profile across the Sea of Marmara and provides a good estimate of the pre-kinematic, average crustal structure.The 1D velocity model calculated by

Tary et al. [2011] is a composite model based on: i) high-resolution wide angle OBS data from the Tekirdag basin for the first 4km reported in *Tary et al [2011]*; and ii) the seismic structure derived by *Bécel et al [2009]* for depth > 4 km. Finally, a smooth model composed of the three others models was calculated to combine the informations from the different 1D velocity models (Figure A2-4).

Locations calculated using these four different models allow us to select one model that was well adapted for this network and for the earthquakes detected within the recording period. The best results were obtained using the 1D velocity model of Tary et al. [2011]. This model was thus used hereafter, whenever a 1D model was necessary.

One-dimensional models however do not account for the lateral variability of bathymetry and velocity structure. To encompass these complexities in the calculation of earthquake locations, different 3D-velocity models were used.

First, the 3D tomographic model of Bayrakci et al. [2013] having a grid spacing of 6km/6km/2km was used (Figure A2-5). The pre-kinematic basement (associated to a velocity of 4.2km/s) can be extracted from the model and is observed at a depth of 6.5 km for its deepest part in the Western high region. However, the relatively large grid spacing considerably smooths the effect of bathymetry. In order to correct for bathymetry and properly account for the effect of basin geometry, high-resolution, 3D-velocity models were built for the 20 km x 60 km area covered by the submarine network, using all available geological and geophysical information from the Sea of Marmara (see details in Appendix 2). This information includes, most particularly: i) high-resolution multibeam bathymetric grids *[Le Pichon et al., 2001];* ii) seismic velocity grids based on 3D, crustal tomography *[Bayracki et al., 2013]* and on 2D, wide-angle seismics *[Bécel et al., 2009*]; iii) fault mapping and basin geometry line-drawing, based on the interpretation of all existing seismic profiles (e.g. *[Şengör et al, 2014)]*.

Two high-resolution models -hereafter named BCC (from Bayrakci, Coutellier, Cros)- were built:

- BCC-1 with grid mesh size of 1500 m x 1500 m x 400 m
- BCC-2 with grid mesh size of 750 m x 750 m x 200 m.

Earthquake location of the M 5.1 earthquake, aftershock sequence: results

This section strictly focuses on the fine characterization of the 3-days long aftershock sequence that followed the Mw 5.1 earthquake which occured on July, 25th, 2011, at 17h57 (Fig. 9). The fine scale characterization was obtained following a two-steps procedure: 1) absolute locations were first obtained using a 3D-velocity model and NLLOC, a non linear routine developed by Lomax (see references); then, relative locations were obtained using HYPODD *[Waldhauser et al, 2000]* and the 1-D velocity model of *Tary et al. [2011].*

Unfortunately, the OBS located above the mainshock hypocenter did not function. As a result, relative relocation, using HypoDD (1D and 3D) *[Waldhauser, 2001]* or NonLinLoc-3D [*Lomax et al, 2012],* was performed with relative distant stations (~15 km). This turned out to be possible only over a limited (~ 20%) number of events, likely due to computation instabilities, which resulted in “air” or “water column” relocations for the most shallow events (see also Appendix 4). This result does not prove, but is consistent with the existence of an important proportion of shallow events.

Also note that, for the events associated to this aftershock sequence, the number of P-arrivals picks is not evenly distributed from one OBS to the other (Figure A2-6). Thus, we hereafter consider the results from two sub-networks: i) the complete network, except OBS 4 (located far from the network center); ii) a specific sub-network defined by 4 stations symmetrically distributed on a circle more or less centered on the mainshock: 3 IFREMER OBSs *(OBS1*, *OBS6* and *OBS3)*, and one KOERI station *(KOERI4*). In order to check for the existence of possible artifacts related to the uneven distribution of OBSs, we considered only those events having arrivals picks on these 4 OBSs.

Locations using the 6 km x 6 km x 2 km 3D-velocity model of Bayrakci et al [2013] (Figs. A2-7 and A2-8).

Absolute locations are evenly distributed between the sea-floor and 15 km at depth, with most of the seismicity concentrated on the Western High (Figure A2-7 top). Still, many aftershocks are located away from the mainshock and delineate an E-W trend below the Tekirdag Basin. Relative localization using HypoDD strongly reduces the RMS and focuses the seismicity along the fault on top of the Western High. Still, many events are found in the Tekirdag Basin along the same E-W trend (Figure A2-7 middle). In the vertical section (Figure A2-7 bottom), relative locations appear distributed into 2 groups of events, between the sea-floor and 6 km depth, and between 6 and 12 km depth, respectively.

If one considers only those events having arrival picks on the 4 stations symmetrically distributed around the mainshock (e.g. OBS1, OBS6, OBS3 and KOERI4), most absolute locations appear to be mostly focused on the Western High and located at a depth above 15 km (Figure A2-8 top). Relative localization using HypoDD results in enhanced focusing above the Western High with shallower events (depth < 10 km). Interestingly, two groups of earthquakes appear: one with depths between 0 and 5 km; and one with depths between 8 and 14 km.

Locations using the Bayrakci-Coutellier-Cros 3D-velocity model with grid mesh size of 1500 m x 1500 m x 400 m (BCC-1 model) (Figs. A2-9 and A2-10).

Absolute locations are dispersed within the Tekirdag Basin, with E-W trends (Fig. A2-9 top). Relative locations obtained with HypoDD are more focused near the epicenter of the mainshock (Fig. A2-9 middle) and shallower (Fig. A2-9 bottom) than with the Bayrakci's model.

If one considers only those events having arrival picks on the 4 stations symmetrically distributed around the mainshock (e.g. OBS1, OBS6, OBS3 and KOERI4), absolute and relative locations appear to be much more focused and closer from the fault (Figure A2-10 top and middle) than those obtained with the Bayrakci et al. [2013] model (Figure A2-8 top and middle). Two different groups of events clearly appear: one with relative location depths between 0 and 5 km; and one with relative location depths between 8 and 14 km (Fig. A2-10).

Location using the Bayrakci-Coutellier-Cros 3D-velocity model with grid mesh size of 750 m x 750 m x 200 m (BCC-2 model) (Fig. A2-11 and A2-12).

The general trends described above (with BCC-1 model) are enhanced when using the BBC-2 model. Absolute locations are dispersed within the Tekirdag Basin, with E-W trends (Fig. A2-11 top). Relative locations obtained with HypoDD are more focused near the epicenter of the mainshock (Fig. A2-11 middle) and shallower (Fig. A2-11 bottom) than with the Bayrakci's or the BCC-1 model.

If one considers only those events having arrival picks on the 4 stations symmetrically distributed around the mainshock (e.g. OBS1, OBS6, OBS3 and KOERI4), both absolute and relative locations appear to be more focused and closer from the fault (Figure A2-12 top and middle) than those obtained with the Bayrakci et al. [2013] and BCC-1 models (respectively Fig. A2-8 and A2-9, top and middle). Again, two different groups of events clearly appear: one with relative location depths between 0 and 5 km; and one with relative location depths between 8 and 14 km (Fig. A2-12 bottom to be compared with Fig. A2-10 bottom and Fig. A2-8 bottom).

REFERENCES

Bécel, A. (2006). Structure sismique de la Faille Nord Anatolienne en Mer de Marmara, *PhD Thesis, Université Denis Diderot.*

Bécel, A.; Laigle, M.; de Voogd, B.; Hirn, A.; Taymaz, T.; Galvé, A.; Shimamura, H.; Murai, Y.; Lépine, J.-C.; Sapin, M. & Özalaybey, S. (2009). Moho, crustal architecture and deep deformation under the North Marmara Trough, from the SEISMARMARA Leg 1 offshore-onshore reflection-refraction survey, *Tectonophysics* **467**: 1-21.

Bayrakci, G.; Laigle, M.; Bécel, A.; Hirn, A.; Taymaz, T.; Yolsal-Cevikbilen, S. & team, S. (2013). *3-D sediment-basement tomography of the Northern Marmara trough by a dense OBS network at the nodes of a grid of controlled source profiles along the North Anatolian fault*, *Geophys. J. Int.* **194** : 1335-1357

Bohnhoff, M.; Bulut, F.; Dresen, G.; Malin, P. E.; Eken, T. & Aktar, M. (2013). An earthquake gap south of Istanbul, *Nat Commun*. **4:1999**, doi:10.1038/ncomms2999

Cros, E., & Géli, L. (2013), Caracterisation of microseimicity in the Western Sea of Marmara: implications in terms of seismic monitoring (Caractérisation de la micro-sismicité dans la partie occidentale de la faille Nord-Anatolienne en Mer de Marmara : implications en termes de stratégie de surveillance de l'activité sismique), *Project Report, Institut Carnot Ifremer-Edrome, Abondement 2011, N°06/11/2013, 29 pages,* available on-line: http://dx.doi.org/10.13155/38916

Gürbüz, C.; Aktar, M.; Eyidogan, H.; Cisternas, A.; Haessler, H.; Barka, A.; Ergin, M.; Türkelli, N.; Polat, O.; Üçer, S.; Kuleli, S.; Baris, S.; Kaypak, B.; Bekler, T.; Zor, E.; Bicmen, F. & Yoruk, A. (2000). The seismotectonics of the Marmara region (Turkey): results from a microseismic experiment, *Tectonophysics* **316** : 1-17.

Géli, L.; Henry, P.; Zitter, T.; Dupré, S.; Tryon, M.; Cagatay, M.; de Lépinay, B. M.; Le Pichon, X.; Sengor, A.; Gorur, N.; Natalin, B.; Ucarkus, G.; Ozeren, S.; Volker, D.; Gasperini, L.; Burnard, P.; Bourlange, S. & the Marnaut Scientific Party (2008). Gas emissions and active tectonics within the submerged section of the North Anatolian Fault zone in the Sea of Marmara, *Earth Plan. Sci. Lett.* **274** : 34-39.

Hergert, T.; Heidbach, O.; Bécel, A. & Laigle, M. (2011). *Geomechanical model of the Marmara Sea regionaI. 3-D contemporary kinematics*, Geophysical Journal International 185 : 1073-1089.

Hubert-Ferrari, A.; Barka, A.; Jacques, E.; Nalbant, S. S.; Meyer, B.; Armijo, R.; Tapponnier, P. & King, G. C. P. (2000). Seismic hazard in the Marmara Sea region following the 17 August 1999 Izmit earthquake, *Nature* **404** : 269-273.

Karabulut, H.; Schmittbuhl, J.; Özalaybey, S.; Lengliné, O.; Kömeç-Mutlu, A.; Durand, V.; Bouchon, M.; Daniel, G. & Bouin, M. (2011*). Evolution of the seismicity in the eastern Marmara Sea a decade before and after the 17 August 1999 Izmit earthquake*, *Tectonophysics* **510** : 17-27.

Laigle, M.; Becel, A.; de Voogd, B.; Hirn, A.; Taymaz, T. & Ozalaybey, S. (2008). *A first deep seismic survey in the Sea of Marmara: Deep basins and whole crust architecture and evolution*, Earth and Planetary Science Letters 270 : 168-179.

Le Pichon, X.; Sengor, A.; Demirbag, E.; Rangin, C.; Imren, C.; Armijo, R.; Gorur, N.; Cagatay, N.; Mercier de Lepinay, B.; Meyer, B.; Saatcilar, R. & Tok, B. (2001). *The active Main Marmara Fault, Earth Plan. Sci. Lett.*, **192** : 595-616.

Lomax, A., C. Satriano and M. Vassallo (2012), Automatic picker developments and optimization: FilterPicker - a robust, broadband picker for real-time seismic monitoring and earthquake early-warning, Seism. Res. Lett. , **83**, 531-540, doi: 10.1785/gssrl.83.3.531.

Lomax, A. and A. Michelini (2012), Tsunami early warning within 5 minutes, *Pure and Applied Geophysics*, **169**, doi: 10.1007/s00024-012-0512-6.

Lomax, A., A. Michelini, A. Curtis (2009), Earthquake Location, Direct, Global-Search Methods, in *Complexity In Encyclopedia of Complexity and System Science, Part 5, Springer, New York*, pp. 2449-2473, doi:10.1007/978-0-387-30440-3.

Lomax, A., J. Virieux, P. Volant and C. Berge, (2000), Probabilistic earthquake location in 3D and layered models: Introduction of a Metropolis-Gibbs method and comparison with linear locations, in *Advances in Seismic Event Location, Thurber, C.H., and N. Rabinowitz (eds.), Kluwer*, Amsterdam, 101-134

Lomax, A., (2014), Mise en oeuvre et support pour logiciels de traitement automatisé de données sismologiques acquises dans le cadre du projet Européen FP7 Marsite, *Contract report, Reference CNRS MA201301A*, available on line: http://alomax.net/projects/marsite/MA201301A_report_v0.2.pdf

Meade, B. J.; Hager, B. H.; McClusky, S. C.; Reilinger, R. E.; Ergintav, S.; Lenk, O.; Barka, A. & Özener, H. (2002). *Estimates of Seismic Potential in the Marmara Sea Region from Block Models of Secular Deformation Constrained by Global Positioning System Measurements*, *Bull. Seism. Soc. Am.*, **92** : 208-215.

Meghraoui, M.; Aksoy, M. E.; Akyuz, H. S.; Ferry, M.; Dikbas, A. & Altunel, E. (2012). Paleoseismology of the North Anatolian Fault at Güzelköy (Ganos segment, Turkey): Size and recurrence time of earthquake ruptures west of the Sea of Marmara, *Geochemistry, Geophysics, Geosystems* **13**.

Orgülü, G. (2011). *Seismicity and source parameters for small-scale earthquakes along the splays of the North Anatolian Fault (NAF) in the Marmara Sea*, Geophysical Journal International 184 : 385-404.

Lee, W. . K., & Stewart, S. W., 1981. *Principles and applications of microearthquake networks*. Academic Press, .

Tary, J. B.; Géli, L.; Henry, P.; Natalin, B.; Gasperini, L.; Comoglu, M.; Cagatay, N. & Bardainne, T. (2011). Sea-Bottom Observations from the Western Escarpment of the Sea of Marmara, *Bull. Seism. Soc. Am.*, **101** : 775-791.

Tryon, M. D.; Henry, P. & Hilton, D. R. (2012). Quantifying submarine fluid seep activity along the North Anatolian Fault Zone in the Sea of Marmara, *Marine Geology* 315-318 : 15-28.

Waldhauser, F. & Ellsworth, W. L. (2000). A Double-Difference Earthquake Location Algorithm: Method and Application to the Northern Hayward Fault, California, *Bull. Seism. Soc. Am.*, **90** : 1353-1368.

FIGURE CAPTIONS

Fig. A2-1: *Bathymetric map of the Western and Central part of the Sea of Marmara. The study area is delineated by coordinates 40°43'N - 40°54'N – 27°30'E – 28°15'E (OBS 4 and KOERI 2 are not within this study area). Image created with GMT software, Version 4.5.11.*

Fig. A2-2: *Number of events by day recorded on the catalog of the European-Mediterranean Seismological Center (EMSC) (top) and detected by the network of OBS (bottom). Image created with GMT software, Version 4.5.11.*

Fig. A2-3: *Details of the histogram, showing the number of events (over 6 hours periods) detected by the different OBSs, following the Mw 5.1 earthquake that occurred on July 25th, 2011, at 17:57. Almost half of the aftershocks occurred within the first 12 hours after the mainshock. Image created with GMT software, Version 4.5.11.*

Fig. A2-4: *1-D velocity models used in the present study. In black the model of Gurbuz et al. [2000], in green the model of Bécel [2006], in red the model from Tary et al. [2011] and in blue a model calculated from the three others. Image created with GMT software, Version 4.5.11.*

Fig. A2-5: *After Bayrakci et al [2013]. Map view at 2, 4, 6 and 8 km depths of the inversion results. Grid node (black dots) spacing is 6 km x 6 km x 2 km. Grey hexagons are receivers (OBSs and land stations) of the survey. The white points are the considered shots. The white contour (RDE = 0.05) surrounds the well-resolved nodes identified by the checkerboard test. The black contour (DWS = 50) surrounds the nodes, which have been inverted during the inversion. The black crosses are the inverted nodes whereas the red ones are the fixed ones [Bayrakci et al. 2013]. Image created with GMT software, Version 4.5.11.*

Fig. A2-6: *Number of P-arrival picks detected at each OBS (IFREMER and KOERI).Image created with GMT software, Version 4.5.11.*

Fig. A2-7: *Top : Absolute locations of aftershocks using the 3D velocity model of Bayrakci et al. [2013] and NLLOC (e.g. Lomax et al, [2000] ; Lomax et al [2009]). Middle : relative locations using the 1D velocity model of Tary et al. [2011] and hypoDD [Waldhauser et al, 2000]. Bottom : Vertical cross-section showing depth distribution of epicenters (relative locations). Image created with GMT software, Version 4.5.11.*

Fig. A2-8 *In order to check for possible artifacts related to the uneven distribution of OBSs, we*

considered a specific sub-network defined by 4 stations symmetrically distributed on a circle more or less centered on the mainshock : 3 IFREMER OBSs (OBS1, OBS6 and OBS3), and one KOERI station (KOERI4). Only those events having arrivals picks on the 4 OBSs are here considered in the present figure. Top : Absolute locations of aftershocks using the 3D velocity model of Bayrakci et al. [2013] and NLLOC (e.g. Lomax et al, [2000] ; Lomax et al [2009]). Middle : relative locations using the 1D velocity model of Tary et al. [2011] and hypoDD [Waldhauser et al, 2000]. Bottom : Vertical cross-section showing depth distribution of epicenters (relative locations). Image created with GMT software, Version 4.5.11.

Fig. A2-9 *Top : Absolute locations of aftershocks using the BCC-1, 3D velocity model (1.5 km x 1.5 km x 0.4 km) and NLLOC (e.g. Lomax et al, [2000] ; Lomax et al [2009]). Middle : relative locations using the 1D velocity model of Ta (MMF)ry et al. [2011] and hypoDD [Waldhauser et al, 2000]. Bottom : Vertical cross-section showing depth distribution of epicenters (relative locations). Image created with GMT software, Version 4.5.11.*

Fig. A3.10: *In order to check for possible artifacts related to the uneven distribution of OBSs, we considered a specific sub-network defined by 4 stations symmetrically distributed on a circle more or less centered on the mainshock : 3 IFREMER OBSs (OBS1, OBS6 and OBS3), and one KOERI station (KOERI4). Only those events having arrivals picks on the 4 OBSs are here considered in the present figure. Top : Absolute locations of aftershocks using the BCC-1, 3D velocity model (1.5 km x 1.5 km x 0.4 km) and NLLOC (e.g. Lomax et al, [2000] ; Lomax et al [2009]). Middle : relative locations using the 1D velocity model of Tary et al. [2011] and hypoDD [Waldhauser et al, 2000]. Bottom : Vertical cross-section showing depth distribution of epicenters (relative locations).Image created with GMT software, Version 4.5.11.*

Fig. A2-11: *Top : Absolute locations of aftershocks using the BCC-2, 3D velocity model (750 m x 750 m x 200 m) and NLLOC (e.g. Lomax et al, [2000] ; Lomax et al [2009]). Middle : relative locations using the 1D velocity model of Tary et al. [2011] and hypoDD [Waldhauser et al, 2000]. Bottom : Vertical cross-section showing depth distribution of epicenters (relative locations).Image created with GMT software, Version 4.5.11.*

Fig. A2-12: *In order to check for possible artifacts related to the uneven distribution of OBSs, we considered a specific sub-network defined by 4 stations symmetrically distributed on a circle more or less centered on the mainshock : 3 IFREMER OBSs (OBS1, OBS6 and OBS3), and one KOERI station (KOERI4). Only those events having arrivals picks on the 4 OBSs are here considered in the present figure. Top : Absolute locations of aftershocks using the BCC-2, 3D velocity model (750 m x 750 m x 200 m) and NLLOC (e.g. Lomax et al, [2000] ; Lomax et al [2009]). Middle : relative locations using the 1D velocity model of Tary et al. [2011] and hypoDD [Waldhauser et al, 2000]. Bottom : Vertical cross-section showing depth distribution of epicenters (relative locations).Image created with GMT software, Version 4.5.11.*

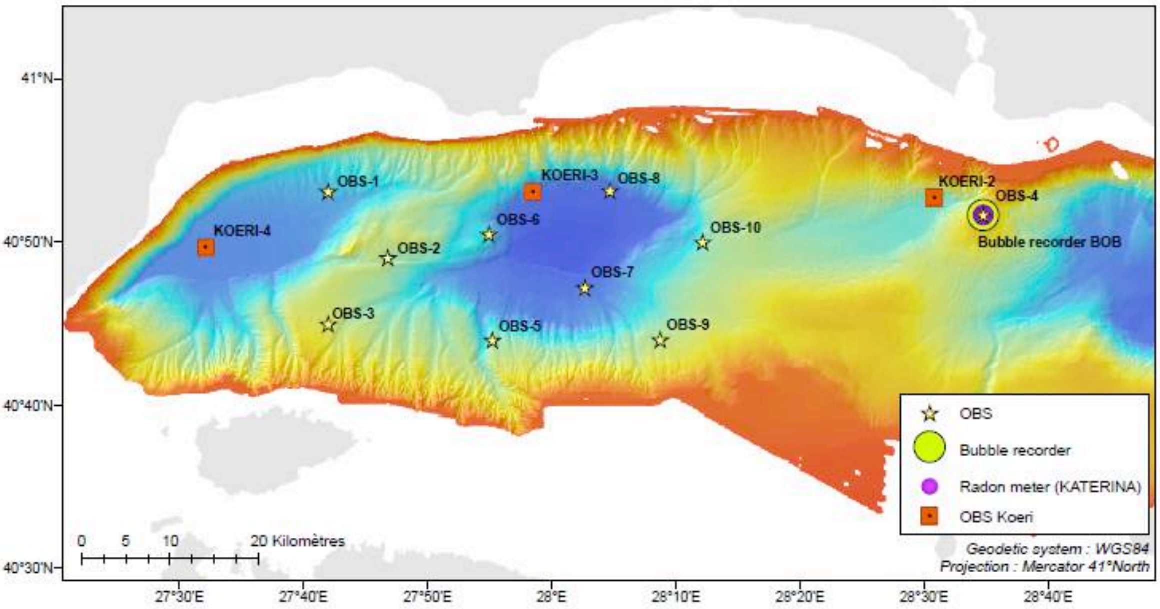

Figure A2-1: *Bathymetric map of the Western and Central part of the Sea of Marmara. The study area is delineated by coordinates 40°43'N - 40°54'N – 27°30'E – 28°15'E (OBS 4 and KOERI 2 are not within this study area). Image created with GMT software, Version 4.5.11.*

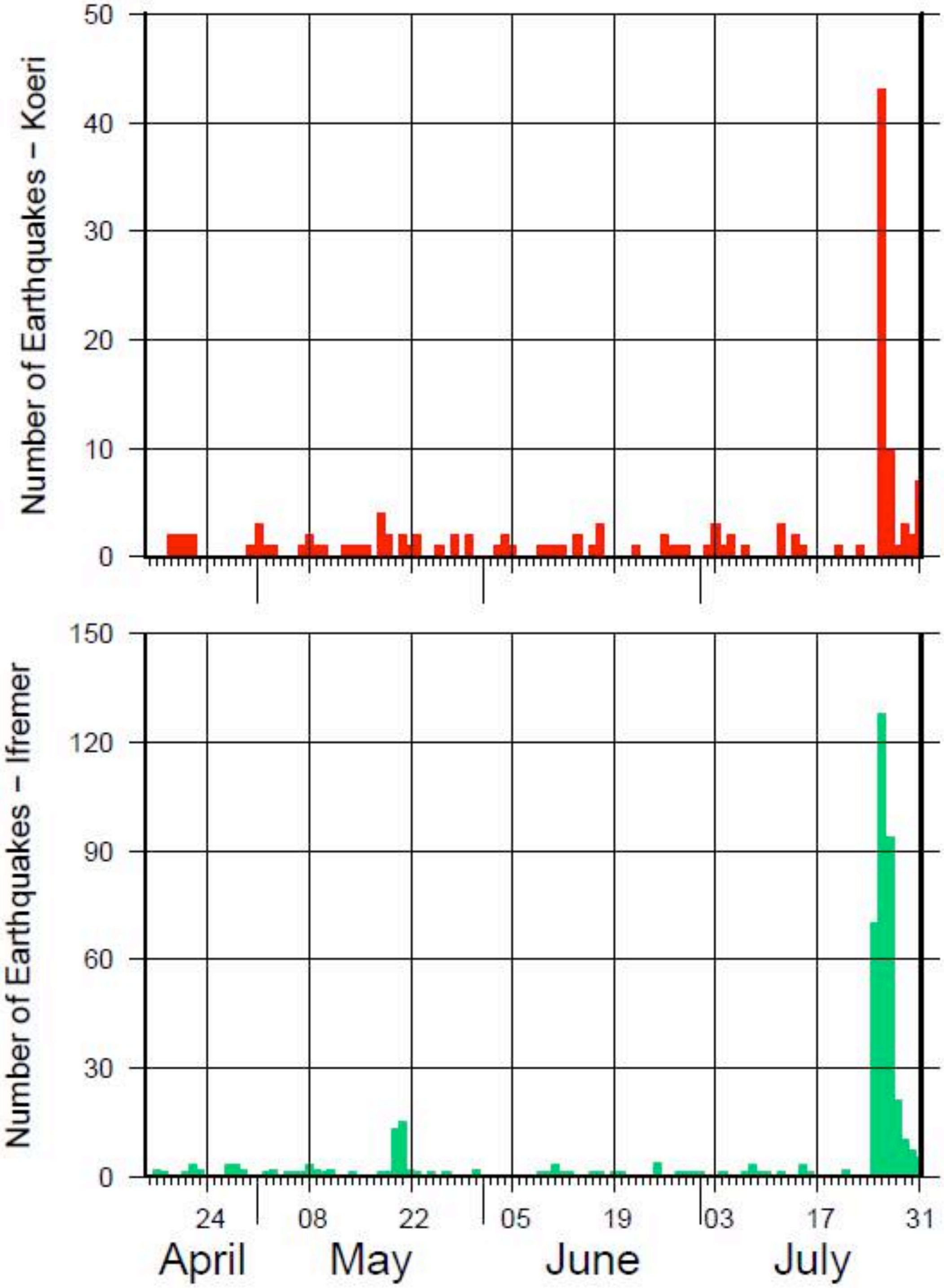

Figure A2-2: Number of events by day recorded on the catalog of the European-Mediterranean Seismological Center (EMSC) (top) and detected by the network of OBS (bottom). *Image created with GMT software, Version 4.5.11.*

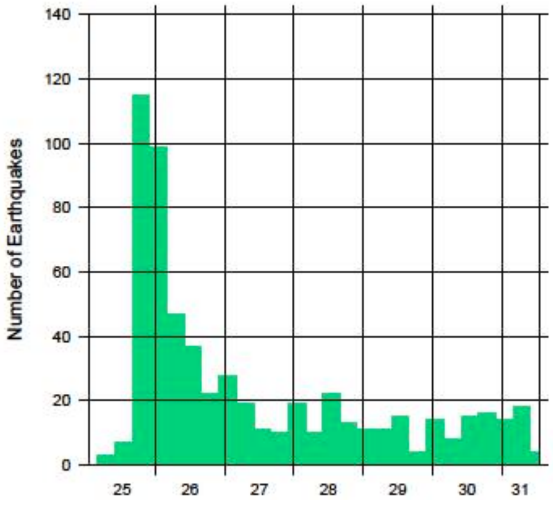

Figure A2-3: Details of the histogram, showing the number of events (over 6 hours periods) detected by the different OBSs, following the Mw 5.1 earthquake that occurred on July 25th, 2011, at 17:57. Almost half of the aftershocks occurred within the first 12 hours after the mainshock. Image created with GMT software, Version 4.5.11.

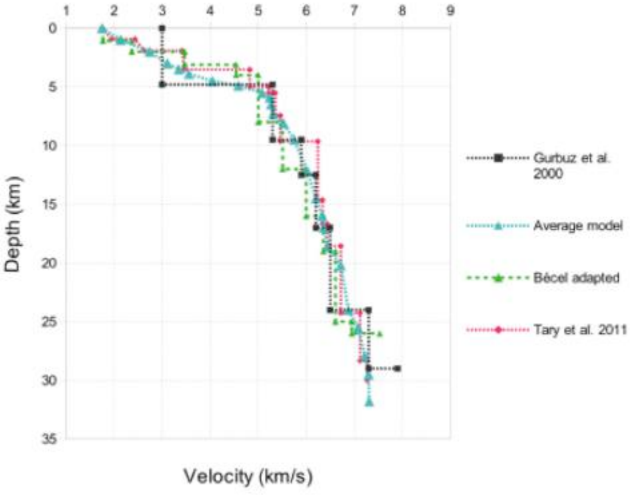

Fig. A2-4: 1-D velocity models used in the present study. In black the model of Gurbuz et al. [2000], in green the model of Bécel [2006], in red the model from Tary et al. [2011] and in blue a model calculated from the three others. Image created with GMT software, Version 4.5.11.

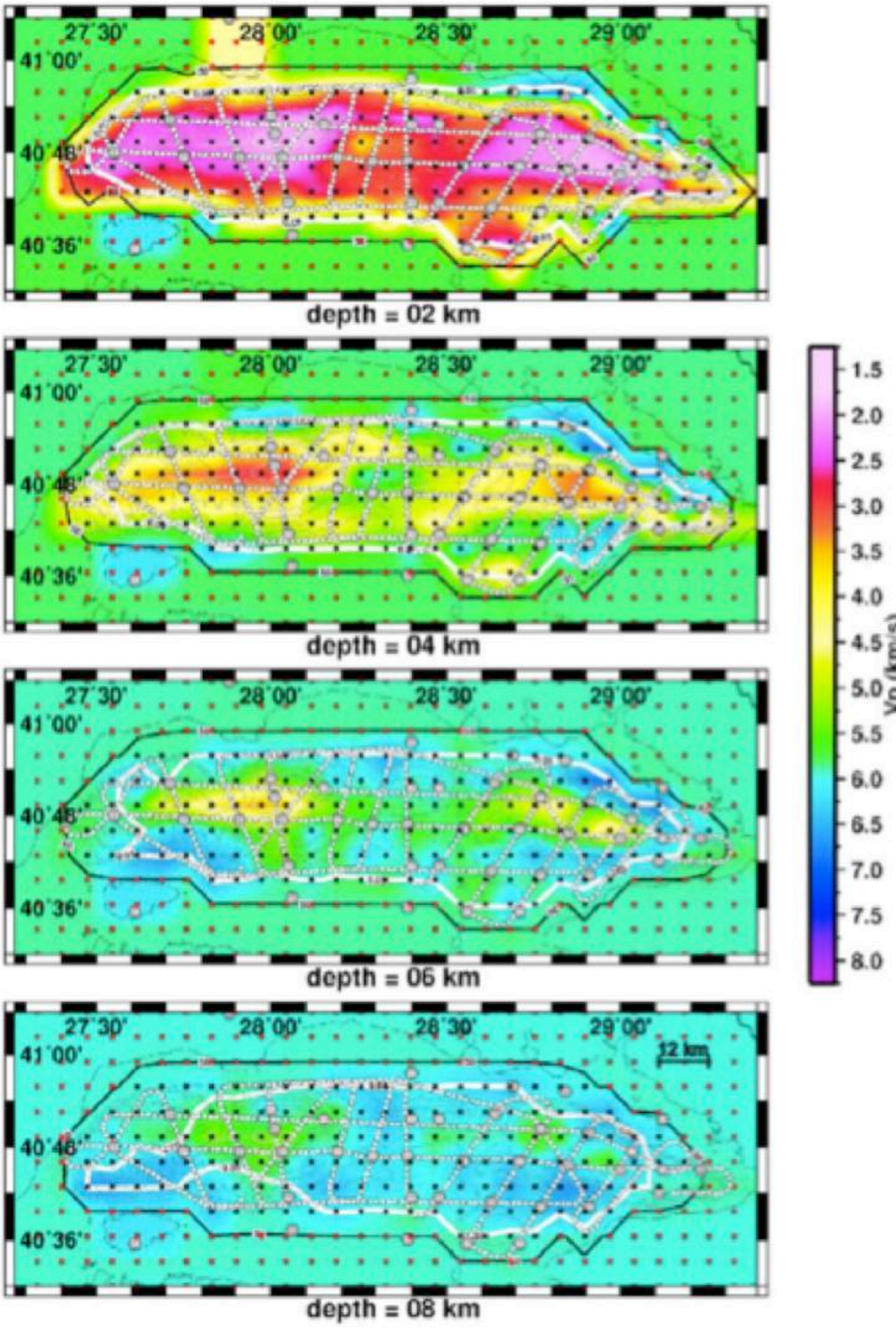

Fig. A2-5: After Bayrakci et al [2013]. Map view at 2, 4, 6 and 8 km depths of the inversion results. Grid node (black dots) spacing is 6 km x 6 km x 2 km. Grey hexagons are receivers (OBSs and land stations) of the survey. The white points are the considered shots. The white contour (RDE = 0.05) surrounds the well-resolved nodes identified by the checkerboard test. The black contour (DWS = 50) surrounds the nodes, which have been inverted during the inversion. The black crosses are the inverted nodes whereas the red ones are the fixed ones [Bayrakci et al. 2013]. Image created with GMT software, Version 4.5.11.

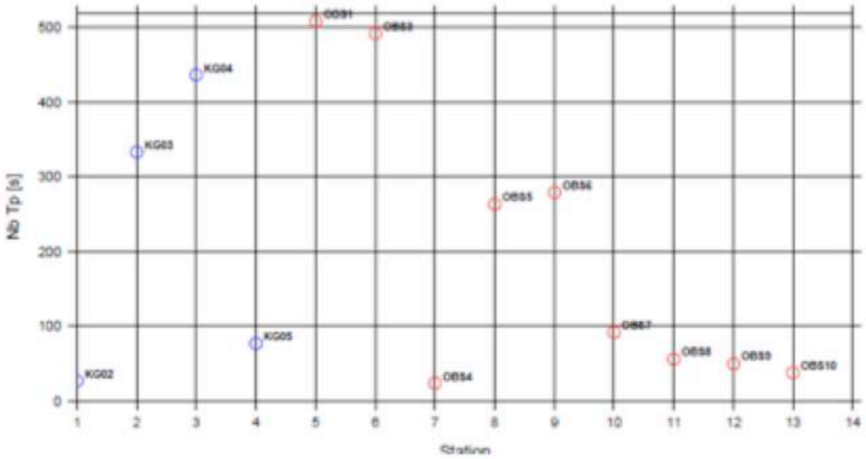

Fig. A2-6 Number of P-arrival picks detected at each OBS (IFREMER and KOERI). Image created with GMT software, Version 4.5.11.

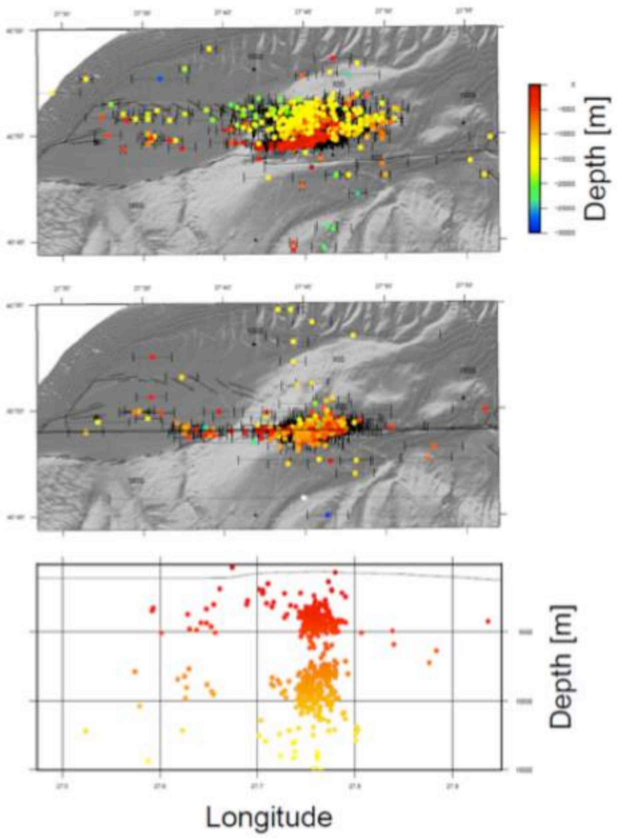

Fig. A2-7: *Top : Absolute locations of aftershocks using the 3D velocity model of Bayrakci et al. [2013] and NLLOC (e.g. Lomax et al, [2000] ; Lomax et al [2009]). Middle : relative locations using the 1D velocity model of Tary et al. [2011] and hypoDD [Waldhauser et al, 2000]. Bottom : Vertical cross-section showing depth distribution of epicenters (relative locations). Image created with GMT software, Version 4.5.11.*

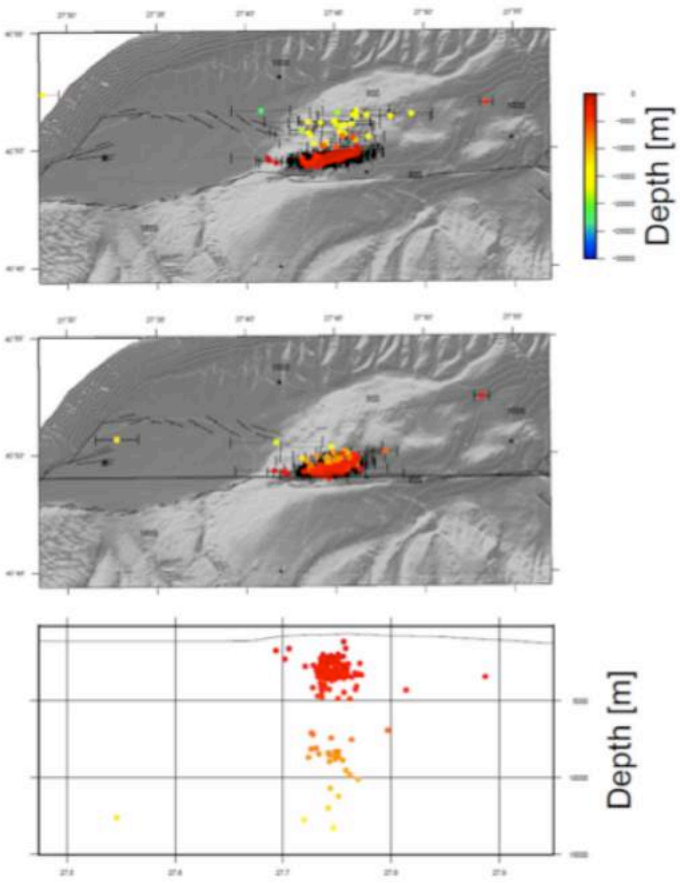

Fig. A2-8 *In order to check for possible artifacts related to the uneven distribution of OBSs, we considered a specific sub-network defined by 4 stations symmetrically distributed on a circle more or less centered on the mainshock : 3 IFREMER OBSs (OBS1, OBS6 and OBS3), and one KOERI station (KOERI4). Only those events having arrivals picks on the 4 OBSs are here considered in the present figure. Top : Absolute locations of aftershocks using the 3D velocity model of Bayrakci et al. [2013] and NLLOC (e.g. Lomax et al, [2000] ; Lomax et al [2009]). Middle : relative locations using the 1D velocity model of Tary et al. [2011] and hypoDD [Waldhauser et al, 2000]. Bottom : Vertical cross-section showing depth distribution of epicenters (relative locations). Image created with GMT software, Version 4.5.11.*

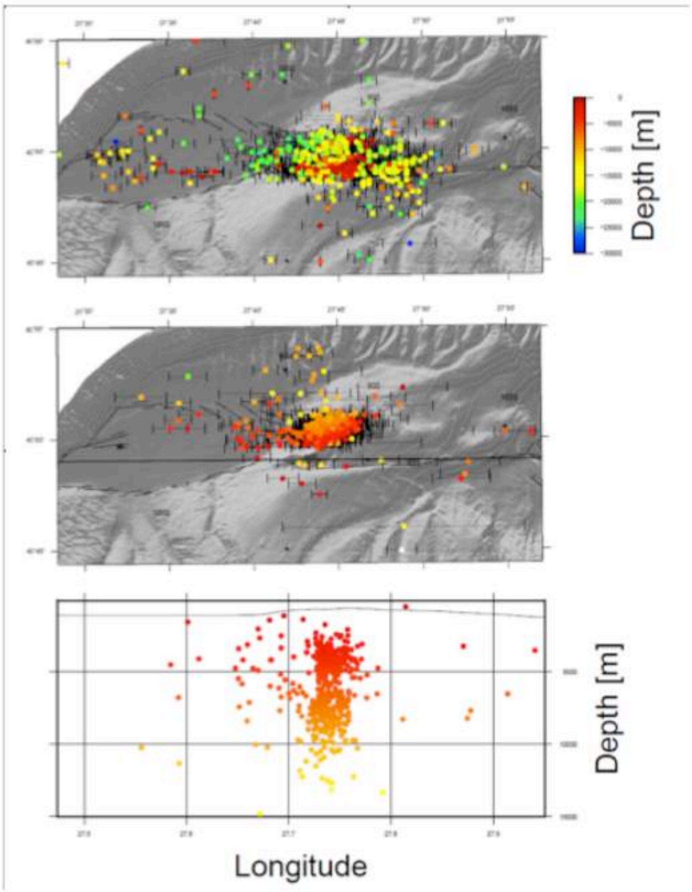

Fig. A2-9 *Top : Absolute locations of aftershocks using the BCC-1, 3D velocity model (1.5 km x 1.5 km x 0.4 km) and NLLOC (e.g. Lomax et al, [2000] ; Lomax et al [2009]). Middle : relative locations using the 1D velocity model of Tary et al. [2011] and hypoDD [Waldhauser et al, 2000]. Bottom : Vertical cross-section showing depth distribution of epicenters (relative locations).Image created with GMT software, Version 4.5.11.*

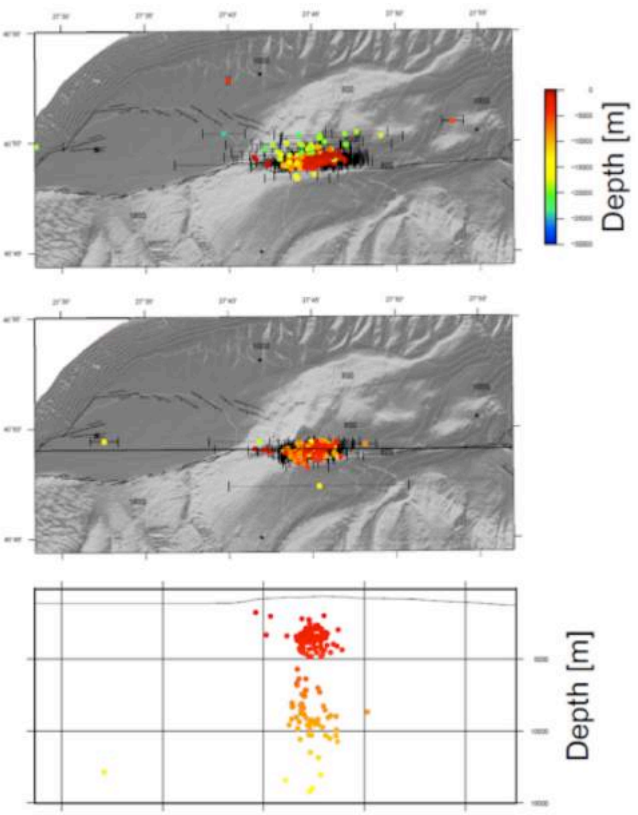

Fig. A2.10: *In order to check for possible artifacts related to the uneven distribution of OBSs, we considered a specific sub-network defined by 4 stations symmetrically distributed on a circle more or less centered on the mainshock : 3 IFREMER OBSs (OBS1, OBS6 and OBS3), and one KOERI station (KOERI4). Only those events having arrivals picks on the 4 OBSs are here considered in the present figure. Top : Absolute locations of aftershocks using the BCC-1, 3D velocity model (1.5 km x 1.5 km x 0.4 km) and NLLOC (e.g. Lomax et al, [2000] ; Lomax et al [2009]). Middle : relative locations using the 1D velocity model of Tary et al. [2011] and hypoDD [Waldhauser et al, 2000]. Bottom : Vertical cross-section showing depth distribution of epicenters (relative locations). Image created with GMT software, Version 4.5.11.*

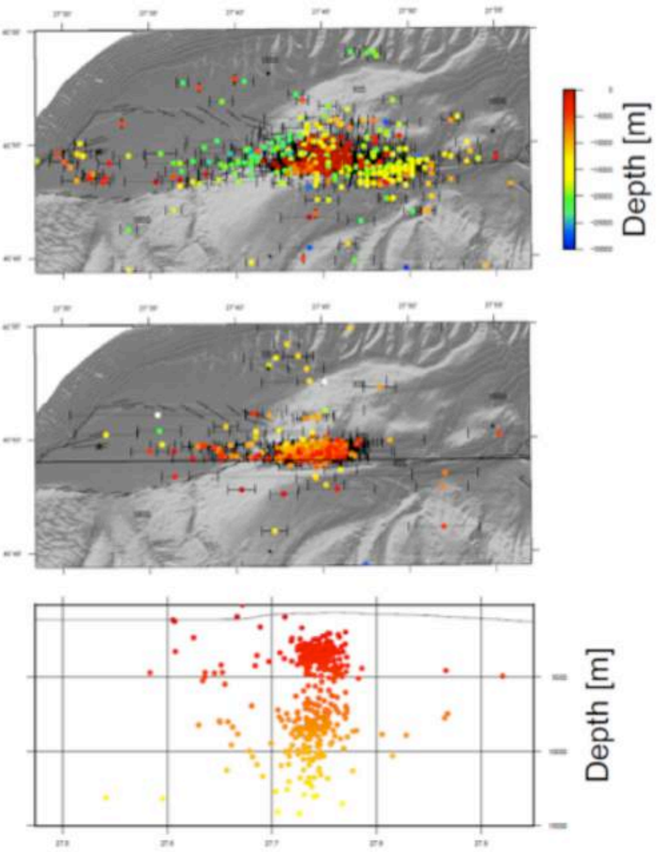

Fig. A2-11: *Top : Absolute locations of aftershocks using the BCC-2, 3D velocity model (750 m x 750 m x 200 m) and NLLOC (e.g. Lomax et al, [2000] ; Lomax et al [2009]). Middle : relative locations using the 1D velocity model of Tary et al. [2011] and hypoDD [Waldhauser et al, 2000]. Bottom : Vertical cross-section showing depth distribution of epicenters (relative locations). Image created with GMT software, Version 4.5.11.*

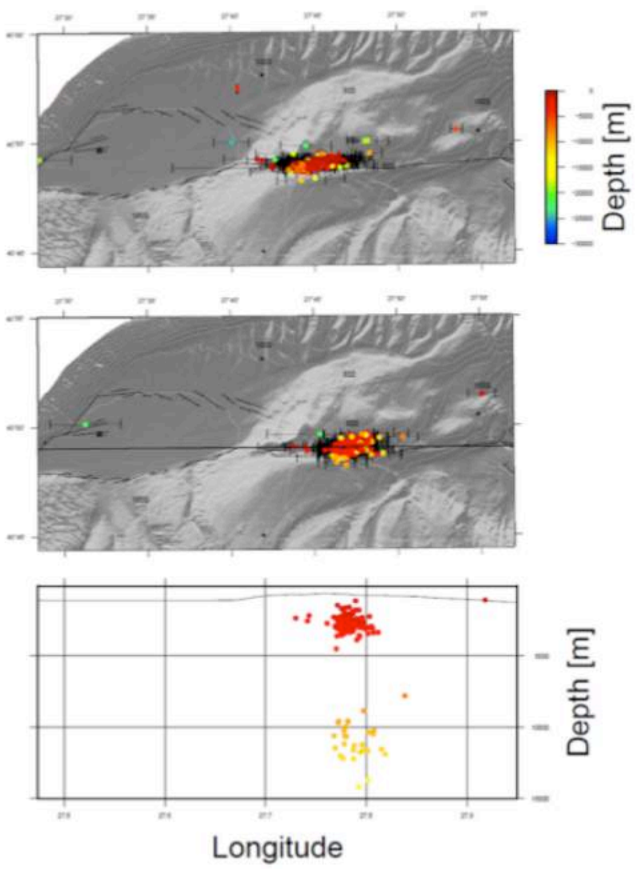

Fig. A2-12: *In order to check for possible artifacts related to the uneven distribution of OBSs, we considered a specific sub-network defined by 4 stations symmetrically distributed on a circle more or less centered on the mainshock : 3 IFREMER OBSs (OBS1, OBS6 and OBS3), and one KOERI station (KOERI4). Only those events having arrivals picks on the 4 OBSs are here considered in the present figure. Top : Absolute locations of aftershocks using the BCC-2, 3D velocity model (750 m x 750 m x 200 m) and NLLOC (e.g. Lomax et al, [2000] ; Lomax et al [2009]). Middle : relative locations using the 1D velocity model of Tary et al. [2011] and hypoDD [Waldhauser et al, 2000]. Bottom : Vertical cross-section showing depth distribution of epicenters (relative locations). Image created with GMT software, Version 4.5.11.*

List of relocated aftershocks triggered by the M 5.1 earthquake of July 25th, 2011 below the Sea of Marmara

Lines finishing with "HASH" indicate those earthquakes that have been used to compute a composite focal mechanism with HASH ùethod (e.g. *[Hardebeck and Shearer, 2003] ; [Hardebeck and Shearer, 2008]*).

References

Hardebeck, J.L. and Shearer, P.M., (2003). Using S/P Amplitude Ratios to Constrain the Focal Mechanisms of Small Earthquakes, Bulletin of the Seismological Society of America, 93, 2434-2444.

Hardebeck, J.L. and Shearer, P.M., (2008). HASH : A Fortran program for computing Earthquake First-Motion Focal Mechanisms -v1.2 – January 31

YY	Mo	DD	HH	min	sec	lat	lon	depth	M_l	ex	ey	ez	
2011	7	25	17	57	33.759	40.819431	27.750807	-11.499000	5.1	0.060000	0.050000	0.227000	
2011	7	25	18	2	42.352	40.821918	27.778866	-0.977988	1.7	0.053000	0.028000	0.233000	
2011	7	25	18	4	34.131	40.818432	27.759413	-2.425820	1.5	0.060000	0.022000	0.260000	
2011	7	25	18	7	41.272	40.821350	27.764523	-6.392250	1.1	0.062000	0.075000	0.227000	
2011	7	25	18	11	3.946	40.816814	27.748871	-2.536190	1.0	0.089000	0.023000	0.143000	
2011	7	25	18	14	44.935	40.816612	27.766193	-2.083400	0.9	0.041000	0.020000	0.133000	
2011	7	25	18	15	27.044	40.816216	27.752365	-2.598190	1.1	0.082000	0.029000	0.271000	
2011	7	25	18	18	24.008	40.820786	27.761404	-0.837845	0.8	0.054000	0.023000	0.110000	
2011	7	25	18	24	21.578	40.817490	27.749113	-6.056920	0.8	0.215000	0.037000	1.189000	
2011	7	25	18	31	25.096	40.818356	27.755905	-0.213655	0.7	0.076000	0.019000	0.068000	
2011	7	25	18	37	49.705	40.818558	27.768202	-2.138260	0.9	0.055000	0.024000	0.226000	
2011	7	25	19	21	46.109	40.824375	27.755228	-7.622220	2.4	0.057000	0.175000	0.713000	
2011	7	25	20	27	55.856	40.816772	27.763325	-0.979471	1.0	0.058000	0.019000	0.050000	
2011	7	25	20	46	19.381	40.821373	27.758614	-6.016800	1.7	0.069000	0.051000	0.169000	
2011	7	25	20	54	42.009	40.817627	27.774149	-0.242670	0.9	0.062000	0.027000	0.068000	
2011	7	25	20	55	11.677	40.815983	27.754362	-0.862758	2.2	0.033000	0.017000	0.080000	
2011	7	25	20	57	7.607	40.815800	27.749414	-4.034590	1.6	0.095000	0.033000	0.452000	HASH
2011	7	25	20	58	59.732	40.816532	27.750210	-2.251200	1.0	0.075000	0.024000	0.351000	
2011	7	25	21	13	26.951	40.791153	27.783297	-0.661734	0.9	0.092000	0.173000	0.137000	
2011	7	25	21	20	52.292	40.820580	27.765345	-0.845829	0.8	0.079000	0.023000	0.149000	
2011	7	25	21	28	35.160	40.816368	27.750235	-4.150620	1.2	0.080000	0.024000	0.421000	HASH
2011	7	25	21	36	2.710	40.818691	27.758835	-0.782731	0.9	0.068000	0.019000	0.087000	
2011	7	25	21	38	27.921	40.815788	27.752163	-1.179000	1.0	0.044000	0.021000	0.219000	
2011	7	25	21	46	51.210	40.818008	27.746834	-1.734070	1.1	0.046000	0.019000	0.116000	
2011	7	25	21	55	51.740	40.815361	27.739273	-4.665650	1.2	0.070000	0.048000	0.355000	HASH
2011	7	25	21	57	44.987	40.820908	27.752178	-0.724823	0.8	0.033000	0.019000	0.082000	
2011	7	25	22	0	37.534	40.814465	27.749849	-2.257610	1.1	0.047000	0.020000	0.117000	
2011	7	25	22	35	1.716	40.814854	27.745348	-2.197140	1.9	0.075000	0.022000	0.214000	
2011	7	25	22	47	9.692	40.821266	27.770475	-0.960408	1.7	0.034000	0.020000	0.100000	
2011	7	25	22	48	34.486	40.816544	27.782555	-10.257900	0.8	0.062000	0.041000	0.29000	
2011	7	25	23	5	39.640	40.817280	27.754457	-0.731253	0.7	0.045000	0.020000	0.145000	
2011	7	25	23	19	26.254	40.819622	27.738045	-6.585730	0.8	0.078000	0.049000	0.245000	
2011	7	25	23	20	20.935	40.816086	27.754702	-1.861580	1.4	0.042000	0.021000	0.151000	
2011	7	25	23	35	42.583	40.819546	27.752638	-0.122316	1.6	0.057000	0.017000	0.069000	
2011	7	25	23	59	9.000	40.820988	27.749704	-0.486965	0.5	0.065000	0.016000	0.079000	
2011	7	26	0	42	39.445	40.818359	27.770784	-0.951129	1.4	0.049000	0.028000	0.197000	
2011	7	26	1	0	12.520	40.816616	27.742247	-4.846300	0.7	0.120000	0.073000	0.321000	HASH
2011	7	26	1	6	43.238	40.817947	27.763597	-1.299030	1.0	0.044000	0.016000	0.081000	
2011	7	26	1	52	50.681	40.817177	27.746067	-1.317560	1.4	0.059000	0.019000	0.221000	
2011	7	26	2	27	12.293	40.825191	27.783472	-7.062270	1.4	0.085000	0.066000	0.219000	
2011	7	26	2	29	43.519	40.821865	27.750734	-5.615620	0.5	0.079000	0.116000	0.492000	HASH
2011	7	26	2	35	49.205	40.823612	27.779015	-0.664657	0.9	0.038000	0.059000	0.369000	
2011	7	26	3	23	0.318	40.817333	27.747803	-0.318697	1.1	0.068000	0.015000	0.101000	

2011	7	26	3	23	7.579	40.818893	27.757208	-1.528590	1.2	0.076000	0.021000	0.353000	
2011	7	26	4	37	34.894	40.817451	27.768347	-0.732141	1.4	0.034000	0.017000	0.063000	
2011	7	26	5	4	21.888	40.818844	27.762892	-0.682499	0.6	0.048000	0.023000	0.099000	
2011	7	26	5	19	0.774	40.826267	27.772551	-2.665670	1.2	0.144000	0.131000	0.540000	
2011	7	26	5	28	19.289	40.839230	28.142277	-17.534100	0.8	1.266000	0.656000	0.881000	
2011	7	26	5	36	47.072	40.818748	27.738325	-5.766200	0.9	0.062000	0.032000	0.268000	HASH
2011	7	26	6	2	39.577	40.820538	27.769218	-1.427060	0.8	0.041000	0.022000	0.196000	
2011	7	26	6	35	33.297	40.816017	27.762562	-3.637100	1.0	0.094000	0.027000	0.590000	
2011	7	26	6	37	57.030	40.817612	27.752043	-1.523800	1.1	0.065000	0.018000	0.130000	
2011	7	26	7	12	44.812	40.819790	27.748047	-2.961600	0.6	0.052000	0.025000	0.135000	
2011	7	26	7	20	4.667	40.813862	27.747988	-2.674630	0.9	0.078000	0.023000	0.177000	
2011	7	26	7	35	3.878	40.813087	27.763771	-0.879162	1.2	0.069000	0.058000	0.286000	
2011	7	26	8	25	58.176	40.823372	27.771366	-5.555770	0.7	0.081000	0.024000	0.418000	HASH
2011	7	26	8	46	29.190	40.816521	27.742756	-2.384820	0.7	0.094000	0.020000	0.159000	
2011	7	26	9	59	41.846	40.820618	27.754610	-2.103570	0.6	0.053000	0.018000	0.201000	
2011	7	26	10	22	46.159	40.825493	27.805195	-0.920796	0.7	0.029000	0.031000	0.153000	
2011	7	26	10	26	19.471	40.819733	27.711847	-0.473366	0.5	0.080000	0.015000	0.063000	
2011	7	26	10	30	21.331	40.816860	27.762030	-0.156155	0.7	0.094000	0.022000	0.055000	
2011	7	26	10	48	9.180	40.817307	27.752171	-0.403453	0.8	0.077000	0.019000	0.088000	
2011	7	26	10	50	5.981	40.818214	27.747742	-7.028020	1.0	0.050000	0.079000	0.178000	
2011	7	26	11	3	52.670	40.821823	27.749489	-6.053760	2.2	0.053000	0.090000	0.249000	
2011	7	26	12	14	23.484	40.814499	27.742170	-6.232930	0.7	0.084000	0.037000	0.336000	
2011	7	26	14	44	42.379	40.843136	27.680277	-25.509300	1.5	0.761000	0.713000	1.39900	
2011	7	26	16	18	17.493	40.815395	27.746065	-0.935856	1.1	0.053000	0.024000	0.214000	
2011	7	26	17	19	36.145	40.820370	27.751101	-1.833300	1.4	0.060000	0.019000	0.310000	
2011	7	26	19	15	17.377	40.815212	27.752506	-1.870670	0.9	0.081000	0.027000	0.313000	
2011	7	26	19	45	28.232	40.829102	27.748108	-14.650300	1.0	0.056000	0.056000	0.21200	
2011	7	26	21	44	28.948	40.816528	27.741415	-0.796585	0.7	0.088000	0.018000	0.121000	
2011	7	26	22	28	34.738	40.823235	27.767639	-2.058960	1.2	0.081000	0.030000	0.133000	
2011	7	26	23	5	49.268	40.822105	27.757240	-4.613740	0.7	0.098000	0.033000	0.424000	HASH
2011	7	26	23	18	57.630	40.821583	27.774755	-0.817410	1.2	0.036000	0.026000	0.114000	
2011	7	27	1	6	53.909	40.820374	27.764826	-1.137760	0.9	0.082000	0.018000	0.104000	
2011	7	27	1	30	49.476	40.814671	27.750582	-3.769950	0.6	0.065000	0.025000	0.266000	
2011	7	27	2	4	8.257	40.819794	27.760338	-1.356690	0.7	0.048000	0.023000	0.174000	
2011	7	27	7	38	16.265	40.818504	27.766407	-0.923795	0.5	0.089000	0.028000	0.243000	
2011	7	27	7	39	30.759	40.817699	27.768108	-0.242922	0.6	0.041000	0.019000	0.081000	
2011	7	27	8	21	35.681	40.819805	27.774345	-0.202807	0.5	0.096000	0.032000	0.095000	
2011	7	27	8	40	22.215	40.816769	27.754026	-1.348200	0.5	0.082000	0.019000	0.150000	
2011	7	27	8	53	19.209	40.826210	27.743307	-6.685130	0.5	0.109000	0.031000	0.171000	
2011	7	27	9	48	39.364	40.818966	27.746773	-0.037935	0.7	0.038000	0.016000	0.030000	
2011	7	27	9	54	55.778	40.820225	27.725117	-14.713900	0.6	0.069000	0.059000	0.231000	
2011	7	27	10	21	54.645	40.819927	27.747383	-4.994490	0.9	0.113000	0.033000	0.489000	HASH
2011	7	27	15	34	32.105	40.823910	27.755653	-0.784939	1.1	0.066000	0.067000	0.306000	
2011	7	27	20	55	47.824	40.815945	27.744890	-3.135910	0.9	0.073000	0.019000	0.148000	
2011	7	27	21	40	38.168	40.819309	27.752707	-0.210890	0.8	0.037000	0.020000	0.089000	
2011	7	27	22	44	15.426	40.821056	27.740309	-2.123820	1.3	0.119000	0.026000	0.446000	
2011	7	28	0	55	14.212	40.816841	27.742762	-1.321120	0.9	0.095000	0.016000	0.139000	
2011	7	28	1	10	40.236	40.823986	27.769501	-0.574948	0.9	0.044000	0.025000	0.150000	
2011	7	28	1	39	14.252	40.816879	27.746952	-0.918418	0.9	0.052000	0.016000	0.132000	
2011	7	28	1	44	50.652	40.816395	27.746264	-2.849680	0.9	0.082000	0.021000	0.246000	
2011	7	28	3	28	23.153	40.817825	27.750534	-0.254746	1.1	0.067000	0.016000	0.070000	
2011	7	28	6	14	54.680	40.814960	27.775223	-7.129040	0.9	0.057000	0.041000	0.191000	
2011	7	28	9	34	6.758	40.822674	27.763832	-0.493470	3.3	0.046000	0.023000	0.060000	
2011	7	28	11	35	18.085	40.819996	27.764313	-1.001330	1.1	0.054000	0.020000	0.076000	
2011	7	28	16	54	30.753	40.816696	27.744074	-3.210350	1.1	0.085000	0.019000	0.101000	
2011	7	29	1	10	10.672	40.814861	27.745739	-2.506140	1.1	0.087000	0.024000	0.221000	
2011	7	29	1	51	46.690	40.818104	27.745768	-6.947060	1.5	0.061000	0.135000	0.330000	
2011	7	29	9	1	59.355	40.816410	27.739592	-4.679300	1.4	0.050000	0.021000	0.186000	HASH
2011	7	29	13	30	48.176	40.817596	27.748293	-5.126970	1.0	0.069000	0.038000	0.318000	HASH

2011	7	29	14	27	43.220	40.819725	27.745876	-7.148010	1.1	0.136000	0.035000	0.503000	
2011	7	29	21	4	22.831	40.820583	27.764042	-0.839774	1.1	0.051000	0.026000	0.250000	
2011	7	30	3	41	55.533	40.822121	27.758406	-1.462720	1.1	0.070000	0.023000	0.175000	
2011	7	30	3	48	36.609	40.823280	27.758135	-1.171310	0.7	0.094000	0.024000	0.196000	
2011	7	30	6	27	5.510	40.826706	27.781101	-0.819320	0.9	0.048000	0.100000	0.315000	
2011	7	30	10	31	48.249	40.815159	27.748770	-2.207350	1.0	0.052000	0.041000	0.238000	
2011	7	30	16	30	53.164	40.815746	27.749296	-4.690450	1.1	0.063000	0.032000	0.253000	HASH
2011	7	30	21	8	35.998	40.820644	27.769365	-0.030947	1.5	0.042000	0.028000	0.029000	
2011	7	31	7	18	16.526	40.823521	27.771490	-0.535226	1.0	0.044000	0.026000	0.101000	

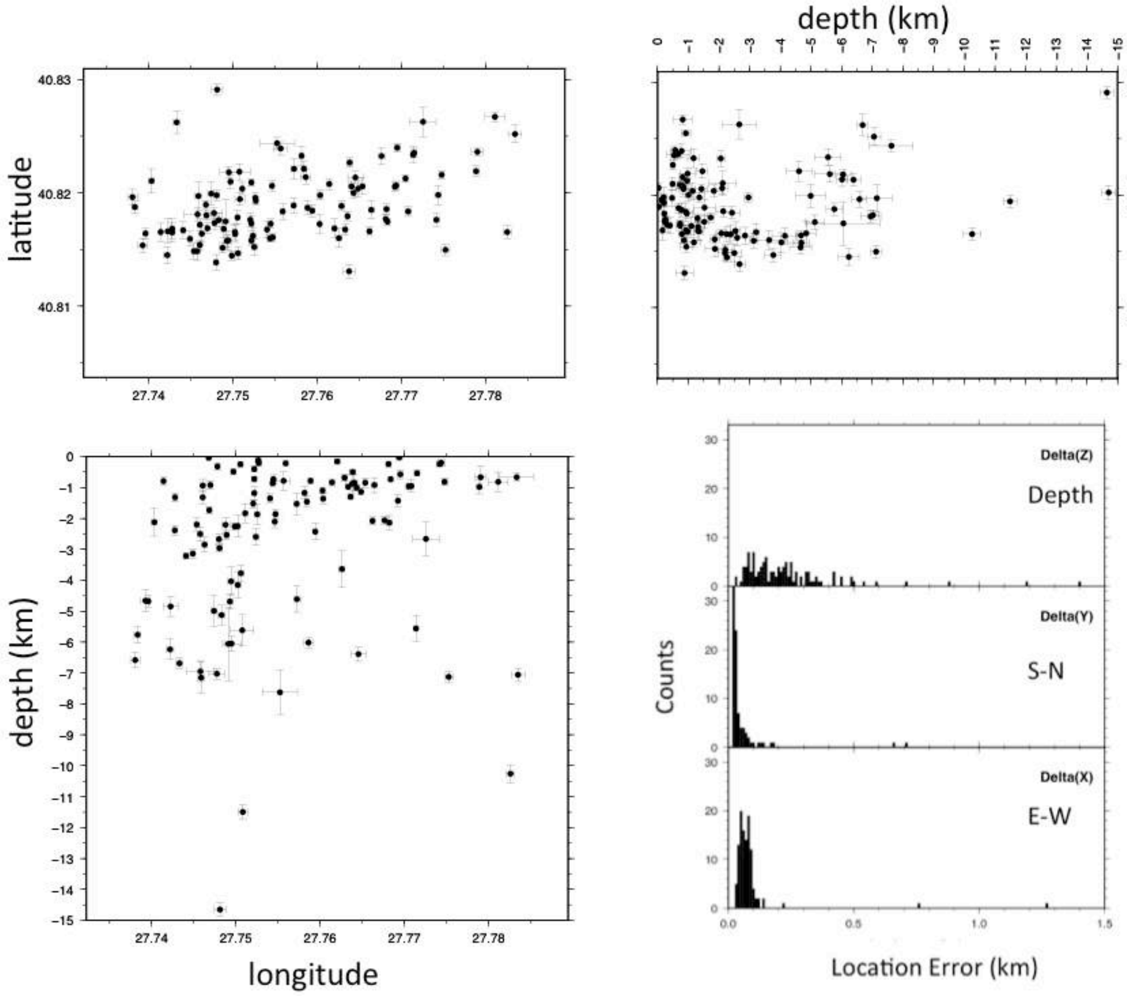

Figure A3-1. Location errors of the above listed aftershocks that followed the July, 25th, 2011 mainshock. a) latitude-longitude plane; b) longitude-depth plane; c) latitude-depth plane. Panel d) displays the distribution of location errors in the 3 directions: depth (deltaz); South-North (delta (y)); Est-West (delta(x)). Image created with GMT software, Version 4.5.11.

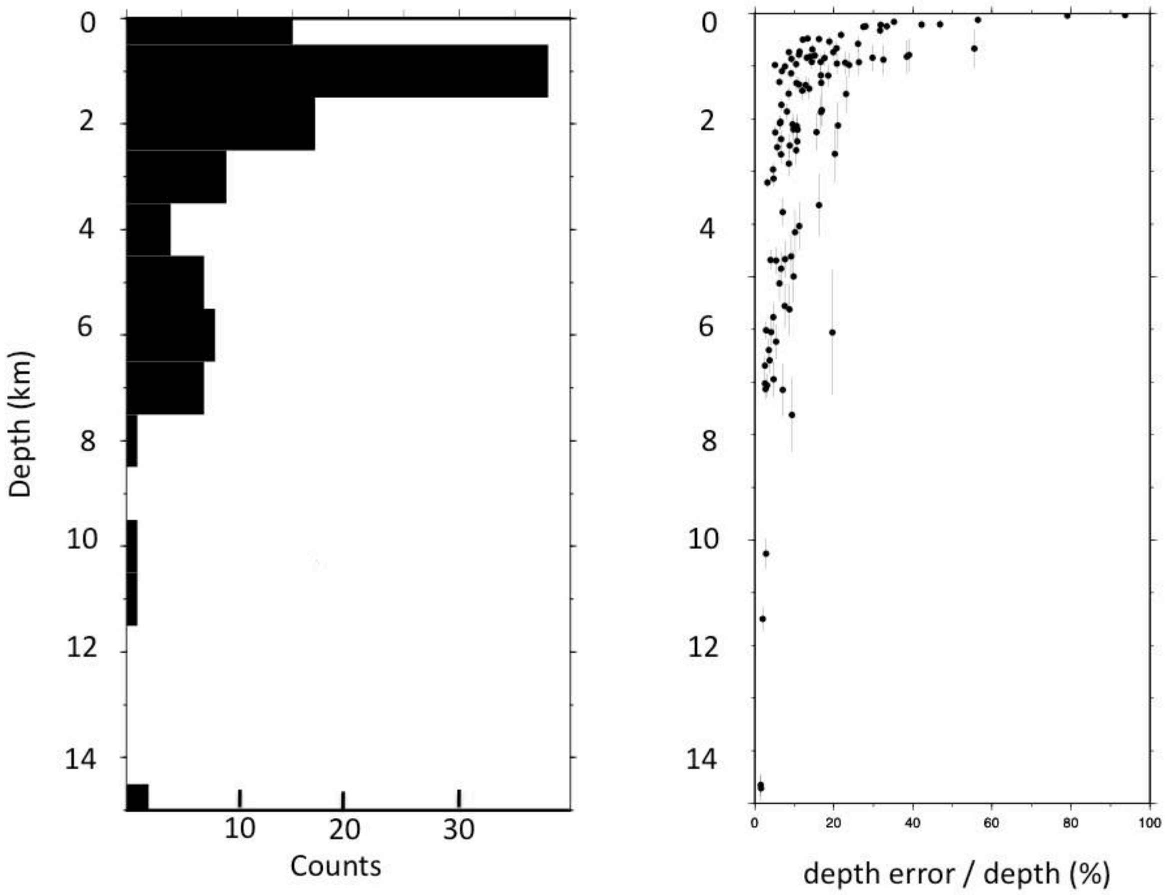

Figure A3-2. Depth distribution of the aftershocks that followed the July, 25th, 2011 mainshock. Right panel indicates the relative depth error, expressed as the ratio between the depth location error and depth, displayed vs depth. Image created with GMT software, Version 4.5.11

APPENDIX 4:
Estimation of geotherm below the Western High area within the Sea of Marmara

Written by Louis Géli, Céline Grall, Pierre Henry

To estimate the depth range at which temperatures ranging between 75°C and 80°C might be expected, eight thermal measurements (Figure A4-1) were obtained from the Western High, where the 3D high-resolution seismic survey was shot: 3 during the Marmesonet Cruise *of R/V Le Suroit* in 2009 and 5 during the *Marm-2010* cruise of R/V Urania in 2010. The measurements were made using autonomous digital temperature probes fitted at different levels on the barrel of a gravity corer. Because of frictional heating produced by the penetration of each probe, the temperature within the sediments was recorded during 7 to 10 min after the penetration and extrapolated to infinite time to yield the ambient, equilibrium temperature in the unperturbed sediment *[e.g., Langseth, 1965]*. Plots of sediment temperature versus depth below seafloor provide thermal profiles (Figure A4-2):

i) within the fault valley, 2 linear profiles, with gradients of respectively, 50.9 $K.km^{-1}$ and 57.8 $K.km^{-1}$, were obtained ;
ii) south of the fault, 2 measurements were made, both at gas emission sites exhibiting linearity and gradient values of 30.7 $K.km^{-1}$ and 35.3 $K.km^{-1}$, respectively
iii) north of the fault, 3 measurements were made: the 2 that were collected a few hundreds of meters away from the mud volcano-like structure exhibit linearity and gradients values ranging between 41.5 $K.km^{-1}$ and 43.1 $K.km^{-1}$; the one profile located from the mound itself departs from linearity (with a meaningless average gradient value of 8.9 $K.km^{-1}$), likely due to the presence of gas and gas hydrates at this site.

Thermal conductivity measurements were performed on cores using the needle probe method *[Von Herzen and Maxwell, 1959]*. Measurements displayed little variability, near 0.84 ± 0.07 $W\ m^{-1}\ K^{-1}$, so the surface heat flow is simply obtained by multiplying the measured thermal gradient by the measured thermal conductivity *[Grall et al, 2012].* The important, observed spatial variability of thermal gradients (Figure A4-2) suggests that the heat transfer to the surface is likely influenced by a variety of processes (including sediment thermal blanketing, fluid circulation, gas hydrate related perturbation, topography etc) that appear difficult to model, mainly due to the scarcity of thermal measurements. To estimate the temperature profile that may be expected, we test different values of basal heat flow (48, 58 and $68.10^{-3}\ W.m^{-2}$), based on the detailed study of the thermal and subsidence history of the Central Basin of *[Grall et al [2012]*. Indeed, the sedimentary column is thick at the Western High but sedimentation rates since at least the last hundred thousand years doesn't reach value higher than 1.5 mm/a *[Grall et al., 2013].* Thus the sediment thermal blanketing should not change drastically the present-day heat flow at the seafloor. Let us consider here: i) that this value (hereafter referred to as Q_b), represents a reasonable proxy for the basal heat flow below the Western High area; and ii) that at the scale of the area, heat flow is conductive, e.g. conservative. Then:
Then:

$$k(z)\frac{dT}{dz} = Q_b \quad (1)$$

which yields :

$$T(z) = T_0 + \int_0^z \frac{Q_b}{k(z)} dz \quad (2)$$

where T(z) and k(z) are temperature and thermal conductivity, respectively. Following *[Pribnow et al, 2000]*, we have tested two different approaches to describe the variation for porosity with depth:

- The linear approach:

$$k(z) = k_0 + Az \quad (3)$$

$$T(z) = T_0 + \frac{Q_b}{A} Log(1 + \frac{Az}{k_0}) \qquad (4)$$

where k_0 and A are the thermal conductivity at the surface (0.83 W K^{-1} m^{-1}) and the thermal conductivity gradient, respectively. Using the compilation of *[Pribnow et al, 2000]*, based on the data collected during Legs 101 to 180 of the Ocean Drilling Programme, we tested different values for A (from 0.4 to 1.4 x 10^{-3} W K^{-1} m^{-2}).

- The "porosity approach", which assumes that k(z) depends on porosity and that porosity exponentially increases with depth due to compaction:

$$\varphi(z) = \varphi_0 e^{-az} \qquad (5)$$

and

$$k(z) = \varphi(z) k_w + (1 - \varphi(z)) k_g \qquad (6)$$

where $\varphi(z)$ and φ_0 are porosity at depth z and at sediment surface, respectively, while a stands for Athy's compaction factor, k_w and k_g for thermal conductivity of seawater and sediment grains. The thermal conductivity of grains (k_g) is derived from surface sediment porosity and conductivity as stated below:

$$k_g = k_0 + \frac{\varphi_0}{1 - \varphi_0}(k_0 - k_w) \qquad (7)$$

Using the above formulae, an analytical expression of temperature is found for integral in (1):

$$\mathrm{T(z)} = \mathrm{T}_0 + \frac{Q_b}{k_g a} Log\left|\frac{k(z)}{k_0}\frac{\varphi_0}{\varphi(z)}\right| \qquad (8)$$

Using bottom water temperature of 14°C, temperatures at depth z are found, based on expressions (4) or (8).

References:

Grall, C., Henry, P., Tezcan, D., Mercier de Lépinay, B., Bécel, Géli, L., Rudkiewicz, J.-L., Zitter, T., Harmegnies, F., (2012), Heat flow in the Sea of Marmara Central Basin, possible implications for the tectonic evolution of the North Anatolian Fault, *Geology*, 40: 3-6, doi:10.1130/G32192.32191.

Grall C., Henry, P., Y. Thomas, G.K. Westbrook, M.N. Çagatay, B. Marsset, H. Saritas, G. Çifçi, L. Géli, Slip rate estimation along the western segment of the Main Marmara Fault over the last 330-430 ka by correlating Mass Transport Deposits, (2013), *Tectonics*, 10.1002/2012TC003255.

Langseth, M., (1965), Langseth, M. G. (1965), Techniques of measuring heat flow through the ocean floor, *in Terrestrial Heat Flow, Geophys. Monogr. Ser.*, vol. 8, edited by W. H. K. Lee, pp. 58–77, AGU, Washington, D. C.

Pribnow, D. F. C., M. Kinoshita, and C. A. Stein (2000), Thermal data collection and heat flow recalculations for ODP legs 101 – 180, *Rep. 0120432, Inst. for Jt. Geosci. Res., Leibniz Inst. for Appl. Geosci.*, Hannover, Germany. Available at http://wwwodp.tamu.edu/publications/heatflow/

Von Herzen, R. P., and A. E. Maxwell (1959), The measurement of thermal conductivity of deep sea sediments by a needle probe method, *J. Geophys. Res.*, **64**, 1557–1563.

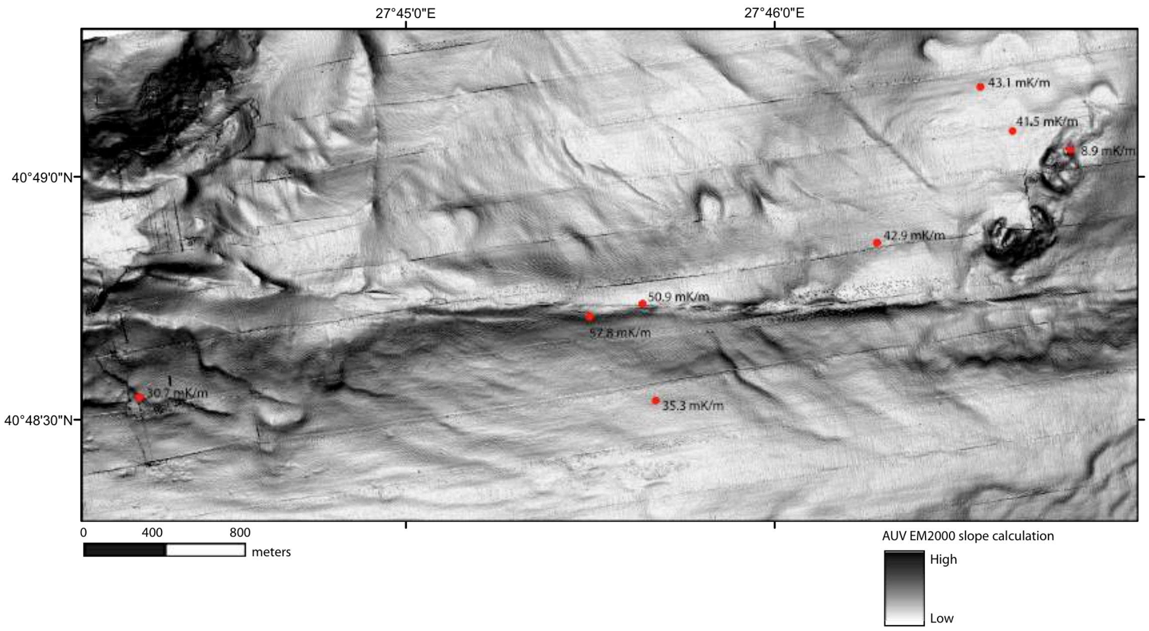

Figure A4-1 : Location (red dots) of thermal measurements performed in 2009 and 2010 in the Western High area. Thermal gradients values are indicated. *Image created with GMT software, Version 4.5.11.*

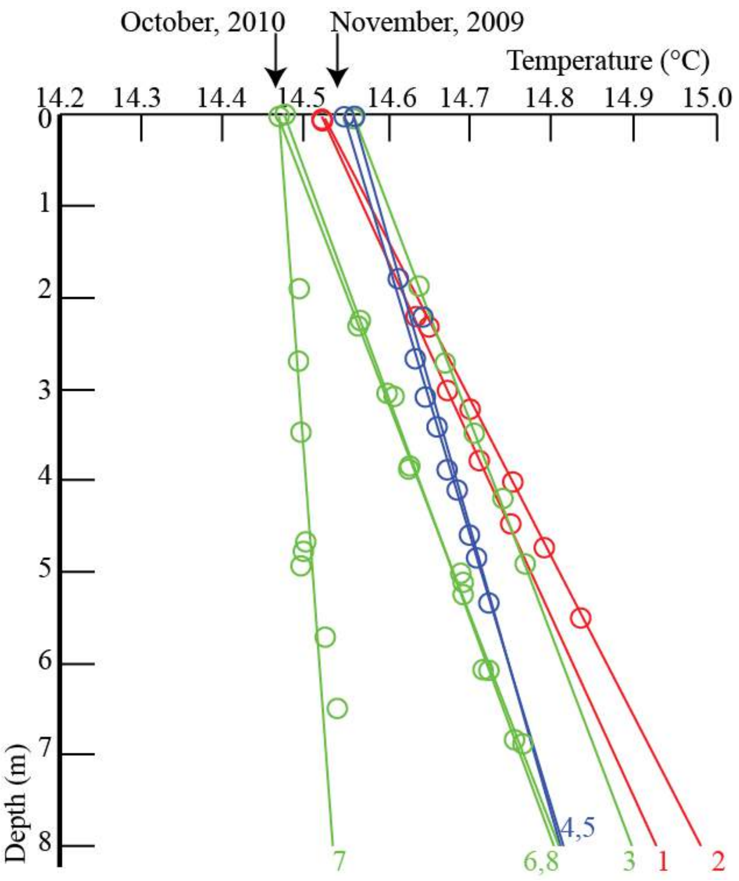

Figure A4-2: Synthesis of the 8 Thermal gradient profiles collected at the Western High. Red profiles refer to measures collected within the fault valley; blue profiles are measures conducted south of the fault and green profiles are the ones acquired north of the Main Fault. Slight differences of seafloor temperature correspond to seasonal variations. Refer to table 1 for linear thermal gradient values deduced and measurement locations. *Image created with GMT software, Version 4.5.11.*

FID	Latitude	Longitude	Thermal Gradient (°C/km)
1	40.811883	27.758283	50.9
2.	40.812316	27.760683	57.8
3.	40.814416	27.771267	42.9
4.	40.809116	27.737950	30.7
5.	40.809000	27.761283	35.3
6.	40.818250	27.777400	41.5
7.	40.817600	27.780016	8.9
8.	40,819750	27,775950	43.1

Table 1: 8 station location and thermal gradient values.

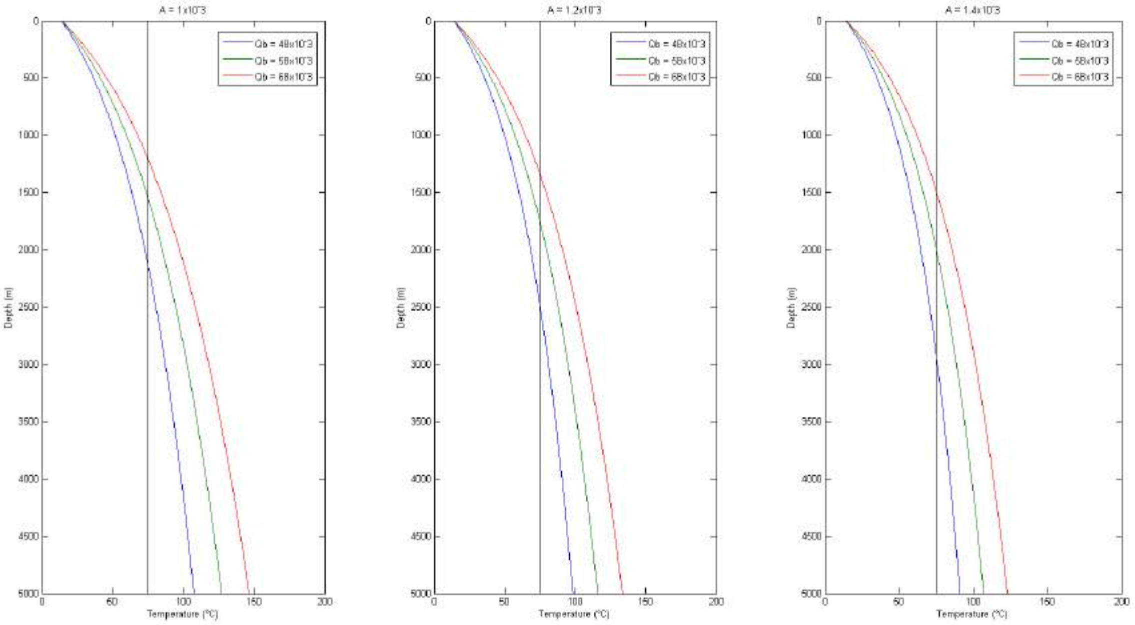

Figure A4-3a : Geotherms computed for different values of input parameters for the model assuming linear variations in thermal conductivity with depth. Thermal conductivity of surface sediments (k_0) is equal to 0.8 W.K^{-1}.m^{-1}. From left to right, A varies from 0.8 x 10^{-3} to 1.2 x 10^{-3} W.K^{-1}.m^{-2}. For each case, different values of basal heat flow are tested (from 48 to 68 mW.m^{-2}). *Image created with GMT software, Version 4.5.11.*

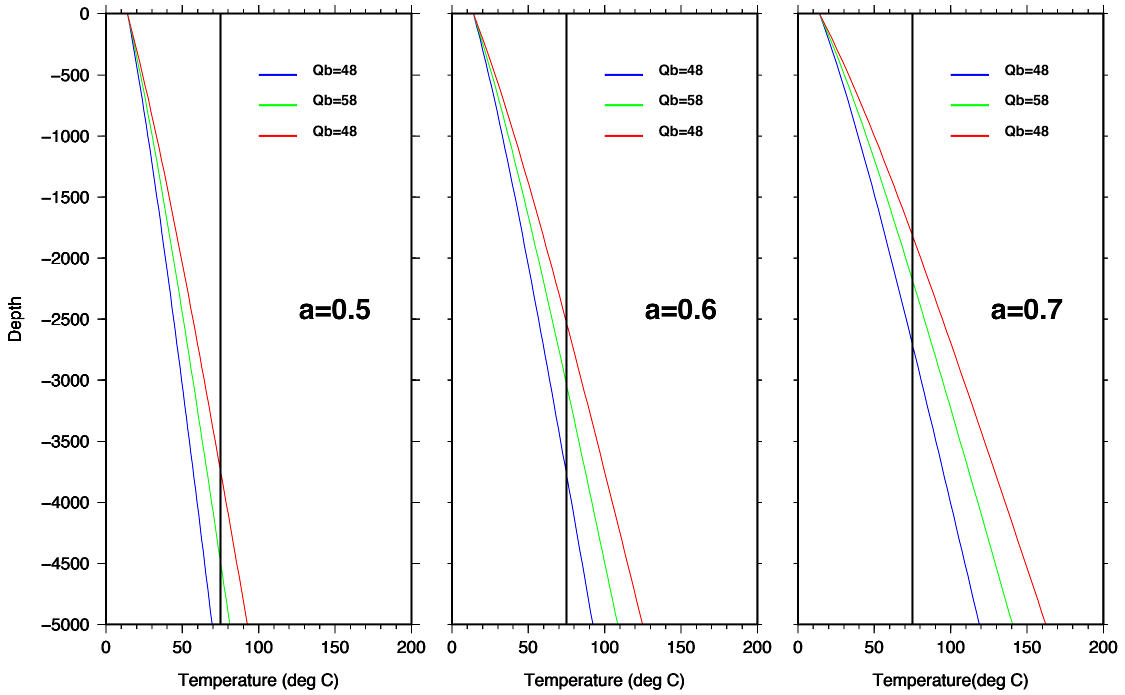

Figure A4-3b: Geotherms computed for different values of input parameters, for the model assuming that thermal conductivity with depth depends on sediment porosity and that porosity exponentially increases with depth. Thermal conductivity (k_0) and porosity (φ_0) of surface sediments are equal to 0.8 $W.K^{-1}.m^{-1}$ and 0.7, respectively. From left to right, the compaction factor is respectively equal to 0.5, 0.6, 0.7 km^{-1}. For each case, different values of basal heat flow are tested (from 48 to 68 $mW.m^{-2}$). *Image created with GMT software, Version 4.5.11.*

APPENDIX 5

High-resolution, 3D- seismic structure of the upper sediments layers below the Western High area

In order to prepare for the implementation of permanent, multiparameter seafloor observatories in the Sea of Marmara, site surveys were conducted within the EU-funded Marmara Demonstration Mission from April 2008 to September 2010. In particular, 3D high-resolution seismic data were collected from the Western High (Figure A5-1), where oil and gas seeps were found, with the objective to image the connections between the fluid migration conduits and the main fault system, a few hundred meters beneath the seabed.

The seismic experiment is described in detail in *[Thomas et al, 2012].* Of particular interest is the existence on the northern side of the NAF of a mound related to a mud-diapir-like structure, having a broadly circular cross-section (Fig. A5-2). This structure is capped by carbonate crusts that pierce the crest of a NE-SW oriented anticline, beneath the location where gas hydrates and hydrocarbons were sampled *[Bourry et al, 2009],* less than 600 m from the NAF main fault trace. It is associated with two other diapirs, aligned NE-SW below the axis of the anticline and deepening away from the fault zone.

Reflections from the seabed above the mound are locally of very strong amplitude and opposite seismic polarity compared to that of the surrounding seabed. This is almost certainly caused by authigenic carbonate outcrops at the seafloor and by the presence of very shallow gas immediately below. This view is coherent by both acoustic surveys and visual observations of gas escaping into the water column and of carbonates at the seabed. All three structures pierce through seismic horizons that are well mapped all over the study area and are characteristically of high amplitude and negative-polarity *[Thomas et al, 2012].* Where horizons are faulted and/or crop out at the seafloor, gas emissions are observed in the water column. In contrast, at unfaulted locations, several horizons appear to collect the gas migrating from depth.

The data also reveal that gas follows buoyancy-driven, upward migration paths in permeable layers and along faults (Fig. A5-3). These paths are controlled by the regional strain field as it is expressed in the seafloor topography, with the primary E-W orientation parallel to NAF and by the secondary tectonic orientations revealed by compressive and extensional features respectively oriented NE-SW and NW-SE *[Grall et al, 2013].* The NAF forms a valley, towards which the adjacent sediment layers are dipping, allowing gas to rise updip, from the valley to the shoulders of the fault. Locally, the conduits of the mud volcano-like structures also offer preferential pathways for the gas to migrate up to the seafloor.

References:

Bourry, C., *et al*, (2009). Free gas and gas hydrates from the Sea of Marmara, Turkey: Chemical and structural characterization. *Chem. Geol.,* doi:10.1016/j.chemgeo.2009.03.007

Dupré S., Scalabrin C., Grall C., Augustin J.-M., Henry P., Görür N., Sengör A. M. C., Cagatay N., Guérin C., Clouet H., Géli L. Tectonic and sedimentary controls for widespread gas emissions in the Sea of Marmara, Results from systematic, ship-borne multibeam echosounder water column imageries, *J. Geophys. Res.*, *Solid Earth*, **120**, doi: 10.1002/2014JB011617 (2015)

Grall, C., et al, (2013) Slip rate estimation along the western segment of the Main Marmara Fault over the last 330 ka by correlating Mass Transport Deposits, *Tectonics*, 10.1002/2012TC003255.

Thomas, Y., *et al*, (2012), Contribution of high-resolution 3D seismic near-seafloor imaging to reservoir scale studies: application to the active north Anatolian Fault, Sea of Marmara, *Near Surface Geophysics*, **10**, 291-301, doi:10.3997/1873-0604.2012019

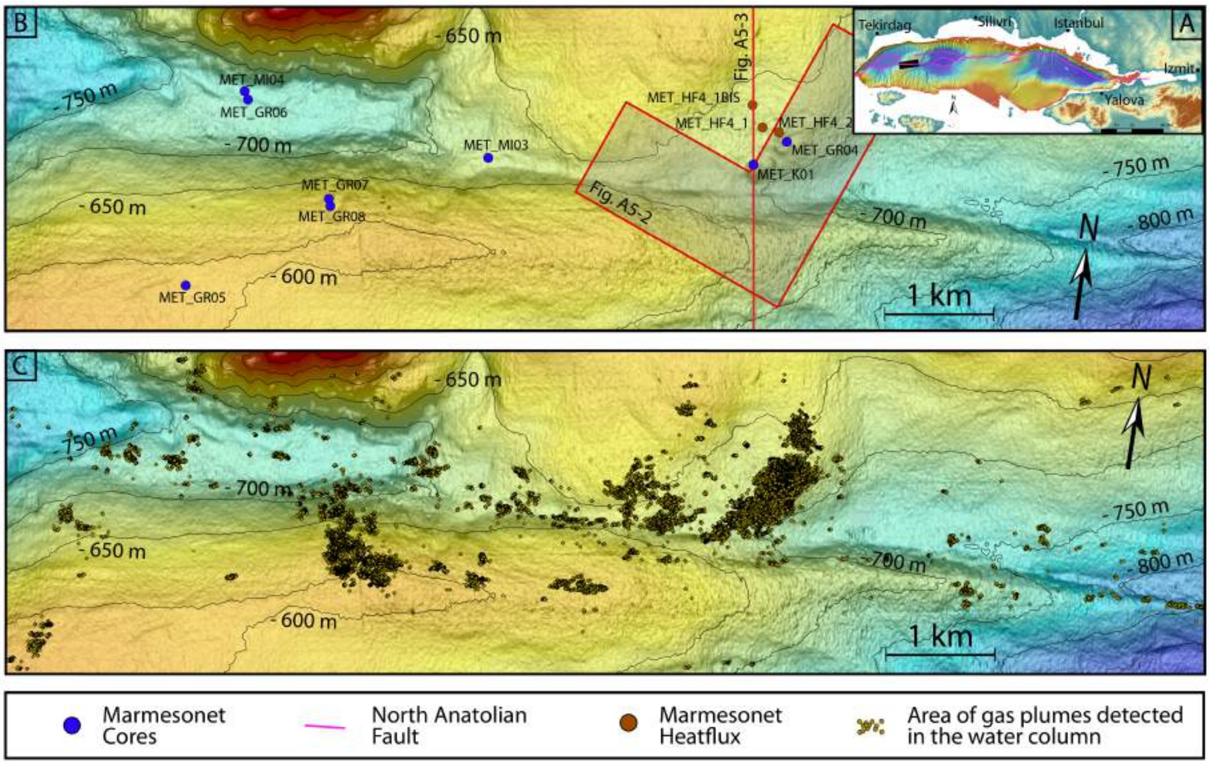

Figure A5-1: A) Inset indicates the location of the area surveyed with High-Resolution 3D seismics *[Thomas et al, 2012]*. B) Detailed bathymetry of the survey area, inferred from HR-seismics, with a lateral resolution of 5m. The shaded area indicates the area represented in Figure A5-2. The North-South red line shows the location of the cross-section shown in Figure A5-3. C) Same as Panel B, with location of gas emissions sites (black dots) detected during the Marmesonet cruise of *R/V Le Suroit* in 2009 *[Dupré et al, 2015].*

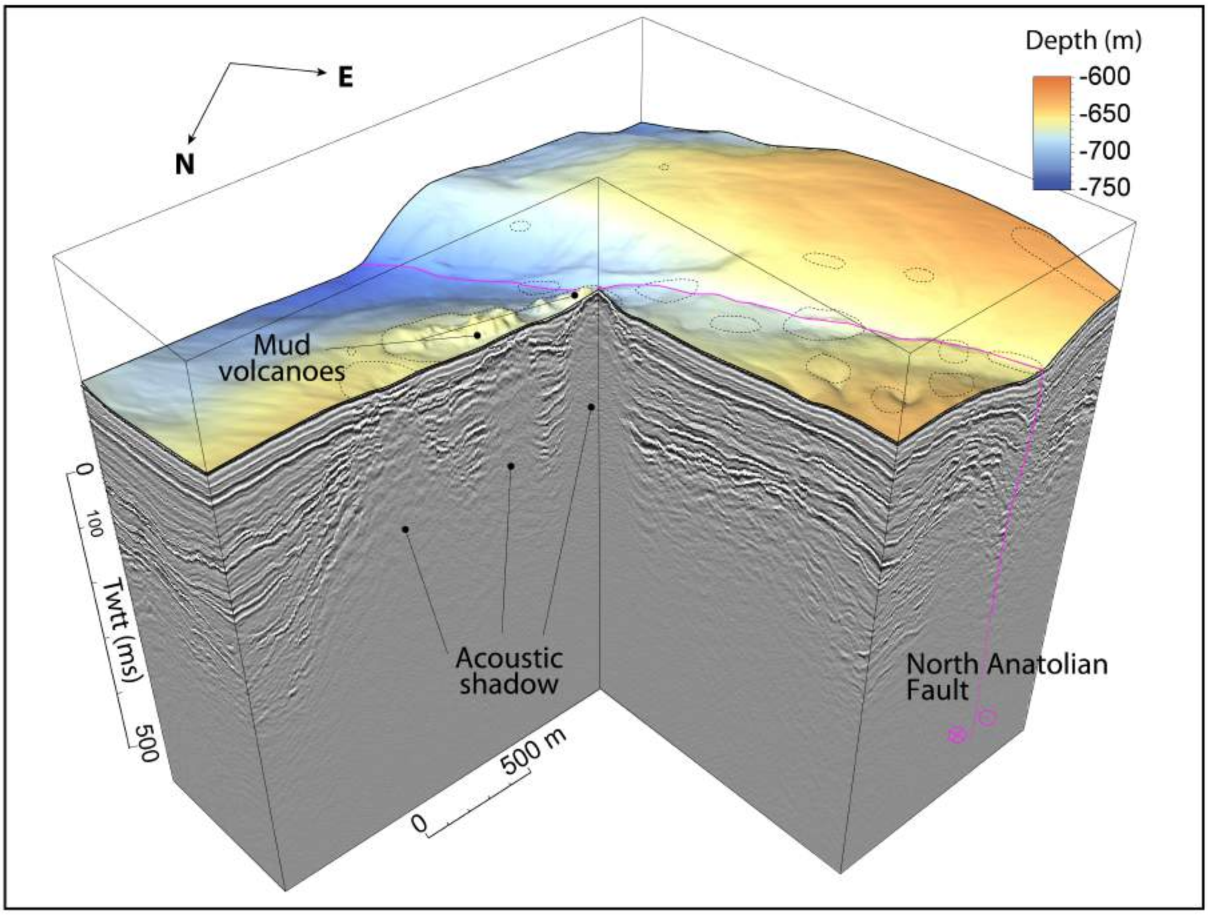

Figure A5-2: Detailed 3D view showing the geometry of the mud-volcano complex relatively to the North-Anatolian Fault Zone (purple line). See details of 3D, HR seismics in *[Thomas et al, 2012].*

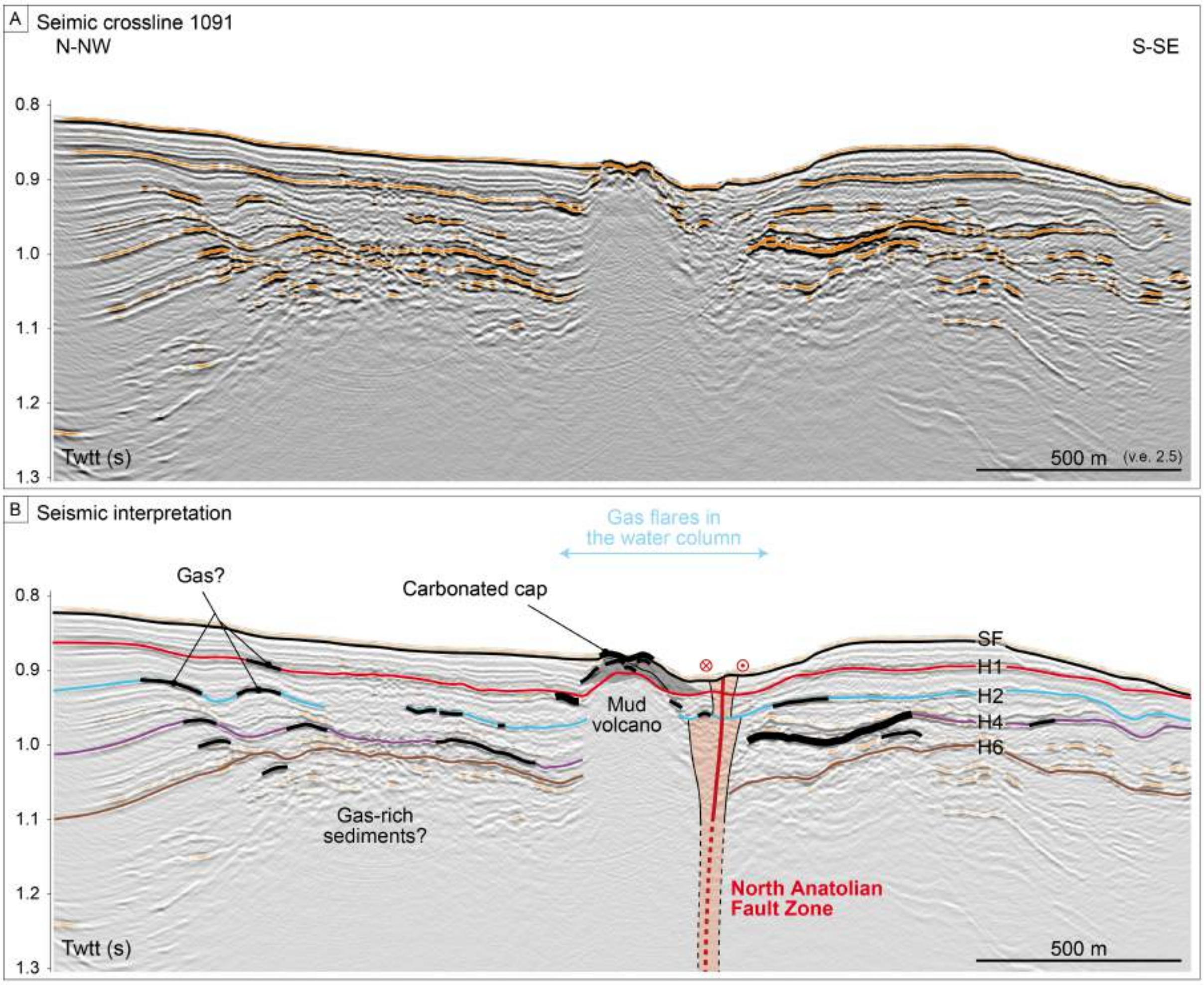

Figure A5-3: Interpreted, North-South section extracted from the HR-3D seismics box (see location in figure A5-1). Reflectors (H1 to H5) are described in *[Grall et al, 2013].*
